# Easy but Efficient: Facile Approach to Molecule with Theoretically Justified Donor–Acceptor Structure for Effective Photothermal Conversion and Intravenous Photothermal Therapy

**DOI:** 10.1002/advs.202309068

**Published:** 2024-03-13

**Authors:** Yuan‐Hui Zhong, Gui‐Feng Huang, Sheng‐Yi Zhao, Lai‐Hon Chung, Hua‐Tang Zhang, Jin‐Hong Zheng, Yi‐Lang Yan, Wen‐Xiu Ni, Jun He

**Affiliations:** ^1^ School of Chemical Engineering and Light Industry Guangdong University of Technology Guangzhou Guangdong 510006 P. R. China; ^2^ Department of Medicinal Chemistry Shantou University Medical College Shantou Guangdong 515041 P. R. China

**Keywords:** antitumor, *CA–RE* reaction, D–A junction, photothermal therapy, twisted molecules

## Abstract

To accelerate the pace in the field of photothermal therapy (PTT), it is urged to develop easily accessible photothermal agents (PTAs) showing high photothermal conversion efficiency (PCE). As a proof‐of‐concept, hereby a conventional strategy is presented to prepare donor–acceptor (D–A) structured PTAs through cycloaddition‐retroelectrocyclization (*CA–RE*) reaction, and the resultant PTAs give high PCE upon near‐infrared (NIR) irradiation. By joint experimental‐theoretical study, these PTAs exhibit prominent D–A structure with strong intramolecular charge transfer (ICT) characteristics and significantly twisting between D and A units which account for the high PCEs. Among them, the **DMA‐TCNQ** exhibits the strongest absorption in NIR range as well as the highest PCE of 91.3% upon irradiation by 760‐nm LED lamp (1.2 W cm^−2^). In vitro and in vivo experimental results revealed that **DMA‐TCNQ** exhibits low dark toxicity and high phototoxicity after IR irradiation along with nude mice tumor inhibition up to 81.0% through intravenous therapy. The findings demonstrate *CA–RE* reaction as a convenient approach to obtain twisted D–A structured PTAs for effective PTT and probably promote the progress of cancer therapies.

## Graphical abstract

A series of NIR absorption D–A structured molecules are prepared via a facile cycloaddition‐retroelectrocyclization (*CA–RE*) reaction. These molecules achieve high photothermal conversion efficiencies (PCEs). Especially, the **DMA‐TCNQ** shows an impressive PCE of 91.3% and demonstrates excellent photothermal anti‐tumor effects in vivo by intravenous administration.

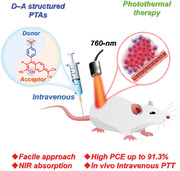

## 1 Introduction

Lung cancer is a significant contributor to cancer‐related mortalities worldwide.^[^ ^1^ ^]^ Although traditional therapies such as chemotherapy and radiotherapy are effective in some cases, they often go along with profound side effects and the treatment effect is always case‐dependent.^[^ ^2^ ^]^ On the other hand, light‐driven phototherapy has been adopted for cancer treatment due to its non‐invasiveness and spatiotemporal control.^[^ ^3^ ^]^ Generally, phototherapy is divided into photodynamic therapy (PDT) and photothermal therapy (PTT). PDT is a clinically established oncologic intervention that relies on the light‐mediated activation of photosensitizers (PSs) to produce reactive oxygen species (ROS).^[^ ^4^ ^]^ PTT has recently emerged as an attractive alternative because of its minimized toxicity and invasiveness in anti‐cancer interventions.^[^ ^5^ ^]^ In PTT, photothermal agents (PTAs) are placed in tumoral tissue to convert NIR light into heat which selectively destroys cancer cells while preserving surrounding normal tissues.^[^ ^6^ ^]^ Even though inorganic materials,^[^ ^7^ ^]^ carbon‐based materials,^[^ ^8^ ^]^ and conjugated polymer nanoparticles^[^ ^9^ ^]^ have been explored as PTAs, their practical uses are hindered by some problems, such as low biocompatibility, poor biodegradability, and high toxicity.^[^ ^10^ ^]^ Therefore, it is necessary to develop PTAs with superior photothermal properties, photostability, and biocompatibility for enhanced clinical performance.

**Scheme 1:**
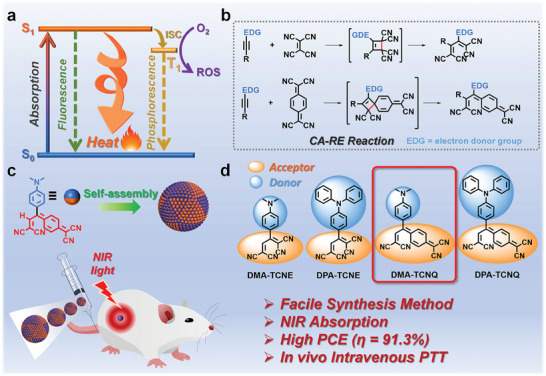
a) Simplified Jablonski energy diagram illustrating the excitation of an electron and subsequent energy relaxation way, b) mechanism of *CA–RE* reaction between electron‐donating substituents on the alkyne reactivity with TCNE or TCNQ, c) schematic illustration of **DMA‐TCNQ** self‐assembly into nanoparticles, and subsequent imaging‐guided PTT in vivo, d) the D–A structured molecules studied in this work.

Considering choosing candidates as PTAs, organic molecules demonstrate some strengths in clinical applications over other types of PTAs in terms of biocompatibility, biodegradability, and long‐term biosafety.^[^ ^11^ ^]^ It is noted that upon photoexcitation, the organic molecule is excited to an excited state (S_1_) followed by deactivation to the ground state (S_0_), primarily through three routes: 1) emitting a photon (i.e., fluorescence), 2) intersystem crossing (i.e., leading to phosphorescence or triplet‐triplet annihilation by other species), and 3) non‐radiative relaxation (heat generation) (**Scheme** **1**a).^[^ ^12^ ^]^ Thus, beyond enhancing one's light absorptivity in the NIR region, suppressing radiative relaxation and promoting non‐radiative relaxation of excited states are also key to improving the photothermal conversion efficiency (PCE) of organic molecules.

Organic molecules with donor–acceptor (D–A) structure manifest excellent light‐harvesting from visible to NIR region through intense intramolecular charge transfer (ICT) from electron‐rich to electron‐deficient moieties.^[^ ^13^ ^]^ Furthermore, the D–A system also benefits quenching of fluorescence and intersystem crossing relaxation, conducive to the photothermal conversion.^[^ ^14^ ^]^ For example, Dong et al. reported the preparation of PTT nanoparticles (NPs) using an organic molecule with an A–D–A structure by reprecipitation.^[^ ^15^ ^]^ These NPs exhibited significant cytotoxicity toward cancer cells in vitro and effectively ablated tumors in vivo. Additionally, they developed a series of D–A functionalized organic PTAs, including furyl‐DPP,^[^ ^16^ ^]^ thienyl‐DPP,^[^ ^17^ ^]^ and selenophene‐DPP,^[^ ^18^ ^]^ which further substantiates the practical potential of PTAs in PTT. At the same time, twisting within the D–A system promotes intramolecular rotational motion and hence non‐radiative transition in excited states, leading to enhanced photothermal conversion.^[^ ^19^ ^]^ Recently, Tang and co‐workers have balanced absorption and quantum yield through precise planar‐twisted molecular engineering for efficient PTT.^[^ ^20^ ^]^ Although there is some progress in D–A structured PTA, the current strategies of prompting PCE of PTAs always involve complex or multi‐step synthesis/modification, such as introducing bulky substituents.^[^ ^21^ ^]^ Meanwhile, another common practice to improve non‐radiative transition is to introduce heavy elements like selenium into the organic motifs, while these elements are somehow harmful to humans, limiting their practical applications.^[^ ^7^, ^18^, ^22^ ^]^ So, it is urged to explore simple ways to prepare PTAs with remarkable D–A structures, low intrinsic toxicity, and competent photodriven cytotoxicity.

The [2+2] cycloaddition‐retroelectrocyclization (*CA–RE*) reaction is one of the simple yet effective approaches to prepare D–A structured molecules, where electron‐donating alkynes form a highly‐strained four‐membered ring with electron‐deficient alkenes followed by ring‐opening to yield a molecule with D–A junction (Scheme 1b). By matching D–A pairs, functionalities, and electronic as well as optical properties, D–A structured target molecules can be designed rationally and tuned systematically through the *CA–RE* reaction (Figure S13, Supporting Information).^[^ ^23^ ^]^ Actually, D–A structures coming from *CA–RE* reaction normally show strong photothermal conversion capacity. Specifically, Gu and co‐workers reported the functionalization of alkyne side chains on the covalent organic framework (COF) by *CA–RE* reaction to yield a radical‐bearing framework for satisfactory photothermal conversion.^[^ ^24^ ^]^ Very recently, He et al. reported facile modification of the main skeleton of alkynes on COF to give a framework holding persistent radicals for high PCE together with solar‐vapor conversion efficiencies > 96%.^[^ ^25^ ^]^ However, there are few studies on the application of D–A structured motifs prepared from *CA–RE* reactions for photothermal conversion while most of the studies focus on non‐linear optics and photoluminescent materials.^[^ ^26^ ^]^ Even though the aforementioned COFs bearing D–A junctions have been adopted for photothermal conversion, these COFs are not nanoscale or insoluble to be utilized for PTT. More importantly, the correlation between PCE and molecular structure is still obscure, hindering the rational design and exploration of efficient PTAs suitable for PTT.

In this regard, we present a series of twisted D–A structured molecules, denoted as **DMA‐TCNE**, **DPA‐TCNE**, **DMA‐TCNQ**, and **DPA‐TCNQ** (Scheme 1d) via facile *CA–RE* reactions. To evaluate whether these molecules are potential PTAs, the electronic structures and properties of these molecules were studied in detail by a joint experimental‐theoretical approach. The results reveal that all of them possess distinct D–A systems resulting in a strong ICT nature, along with non‐zero dihedral angles between D and A units which promotes heat release through non‐radiative deactivation of photoexcited states. It was found that all these molecules exhibit strong absorption in the whole UV–vis spectral range until the NIR region and have high PCEs. Specifically, upon photoirradiation by a 760‐nm LED lamp at 1.2 W cm^−2^, the temperature of **DMA‐TCNQ** (80 µm in 1% DMSO PBS solution) increased from 28.4 to 59.1 °C (Δ*T* = 30.7 °C) within 10 min with PCE of 91.3%. To the best of our knowledge, this is the highest PCE value of organic molecular PTAs. Moreover, by studying the excited state, these molecules execute efficient light‐to‐heat conversion mainly through the rotation of D and A groups. Based on these results, we first evaluated the in vitro anticancer activity of **DMA‐TCNQ**, which was found to show low dark toxicity and a strong tumor cell killing power after 760‐nm LED lamp illumination along with nude mice tumor inhibition up to 81.0% through intravenous therapy. It also demonstrated excellent photothermal anti‐tumor effects in vivo, ablating tumors selectively by intravenous therapy. Overall, our study opens an opportunity to develop photothermally active and photostable PTAs through rational design of D–A junction molecule using the *CA–RE* route.

## 2 Results and Discussion

### 2.1 Theoretical Basis for D–A Structured Molecules

**Figure 1:**
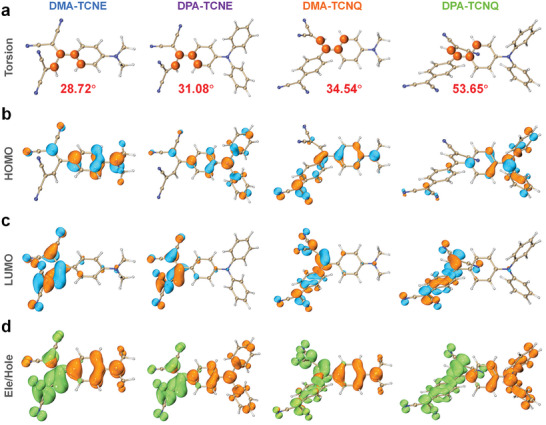
The optimized structures a), the HOMO b) and LUMO c) distributions of **DMA‐TCNE**, **DPA‐TCNE**, **DMA‐TCNQ**, and **DPA‐TCNQ** (the orange region corresponds to a positive value, while the blue region represents a negative value with isovalue of 0.05 au.), the electron‐hole distribution d) of **DMA‐TCNE**, **DPA‐TCNE**, **DMA‐TCNQ**, and **DPA‐TCNQ** (the orange part depicts hole while green part refers to electron with isovalue of 0.002 au.).

Before the preparation of D–A structured PTAs, we first investigated their structural and electronic properties in the ground state through density functional theory (DFT) calculation. The Multiwfn 3.8 and VMD 1.9.3 programs were adopted analyzing orbital composition and visualization, respectively.^[^ ^27^ ^]^ The optimized structure shows that twisting exists between D and A moieties in all these molecules. Specifically, dihedral angles between planes of D and A units for **DMA‐TCNE**, **DPA‐TCNE**, **DMA‐TCNQ**, and **DPA‐TCNQ** are 28.72°, 31.08°, 34.54°, and 53.65° (**Figure** **1**a). Noteworthily, the highest occupied molecular orbitals (HOMOs) are dominantly on D units (DMA or DPA sections) whereas the lowest unoccupied molecular orbitals (LUMOs) are dominated by A units (cyano‐vinyl groups) (Figure 1b,c). Details of the orbital composition of **DMA‐TCNE**, **DPA‐TCNE**, **DMA‐TCNQ**, and **DPA‐TCNQ** are listed in Tables S1–S4 (Supporting Information). These results confirm the strong ICT features of these D–A structured molecules, which correspond to our design strategy and the following experiment. The time‐dependent density functional theory (TD‐DFT) calculation was further conducted to unveil the electronic properties of these D–A structured molecules in the excited state. As shown in Figure S14 (Supporting Information), all these molecules possess strong dipole moment in the S_1_ state, together with dipole moment in **DMA‐TCNQ** (*ρ* = 3.20) larger than other analogs (1.43, 1.80, and 2.69 for **DMA‐TCNE**, **DPA‐TCNE**, and **DPA‐TCNQ**, respectively), indicating the highest degree of charge separation in S_1_ state as well as strongest absorption capacity of **DMA‐TCNQ**. Since electron‐hole analysis is a visual way to reveal the nature of electron excitations,^[^ ^28^ ^]^ electron‐hole distribution of all four D–A structured molecules were calculated and showed that electron density centralizes on A unit while hole density majorly distributes over D unit (Figure 1d) in S_1_ state (fractions of the electron, hole, overlap, and difference listed in Table S5, Supporting Information). The centroids of electrons and holes (*C*_ele_ and *C*_hole_) also confirm the distribution of electrons and holes (Figure S15, Supporting Information). These results consolidate the ICT feature of these molecules.^[^ ^29^ ^]^ On the other hand, the *t*‐index (the degree of electron‐hole separation) was calculated to evaluate the type of charge transfer.^[^ ^28d^ ^]^ The positive values of the *t*‐index signify that all D–A structured molecules studied in this work proceed with charge‐transfer excitation rather than local excitation (Table S6, Supporting Information).

### 2.2 Molecules Preparation and Characterization

**Figure 2:**
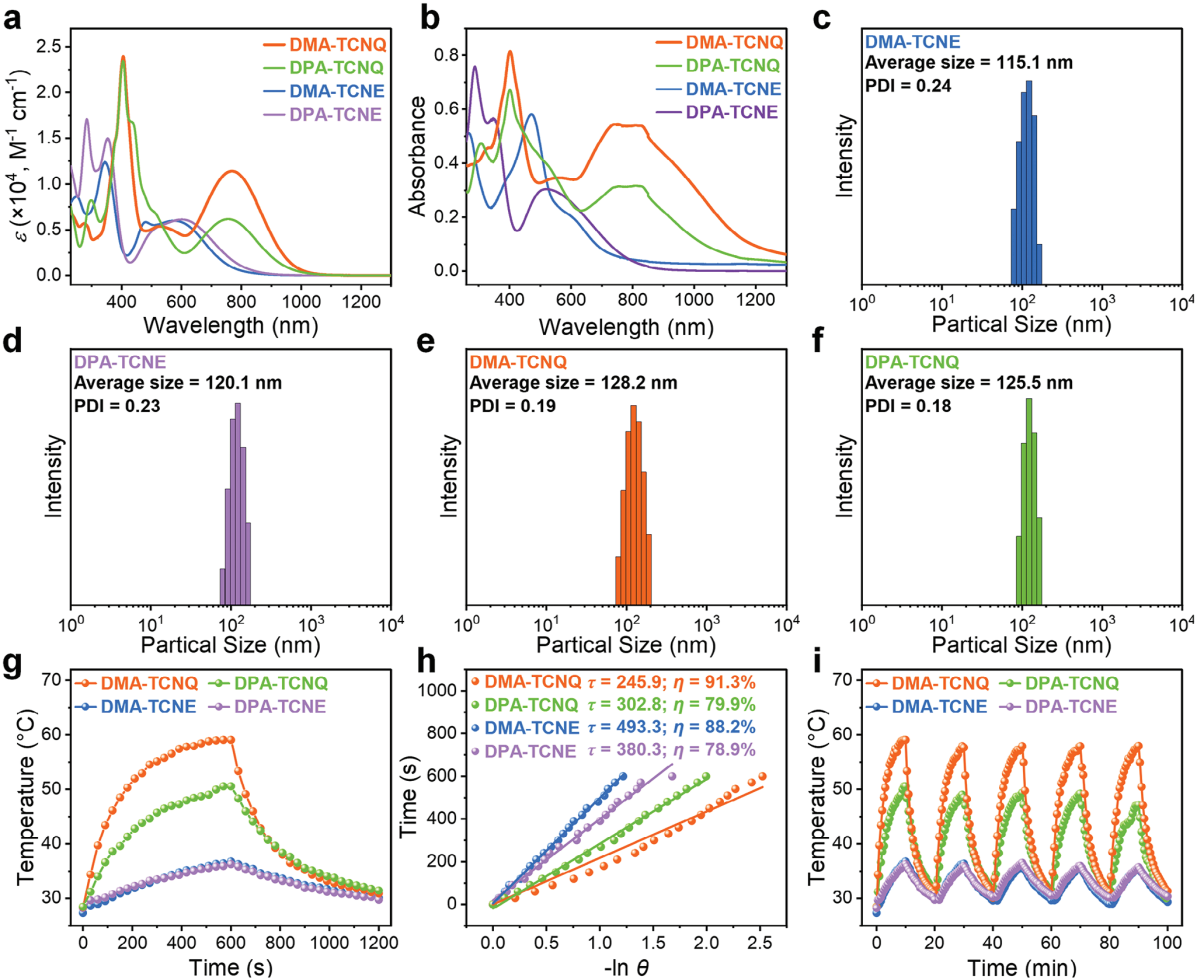
a) The UV–vis–NIR spectra of the D–A structured molecules in DCM (20 µm) and b) in 1% DMSO PBS solution (80 µm); the size distribution of c) **DMA‐TCNE**, d) **DPA‐TCNE**, e) **DMA‐TCNQ**, and f) **DPA‐TCNQ** in 1% DMSO PBS solution measured by DLS, g) heating and cooling curves, and h) linear time data versus −ln*θ* from the cooling period of D–A structured molecules (80 µm in 1% DMSO PBS solution) under 760‐nm LED lamp irradiation (1.2 W cm^−2^), i) temperature elevation of D–A structured molecules (80 µm in 1% DMSO PBS solution) under 760‐nm LED lamp irradiation (1.2 W cm^−2^) for 5 irradiation–cooling cycles.

All four D–A structured molecules (**DMA‐TCNE**, **DPA‐TCNE**, **DMA‐TCNQ**, and **DPA‐TCNQ**) were synthesized in high yields by following modified previously reported methodology involving Sonogashira coupling, desilylation, and a *CA–RE* reaction (for preparation details, see Supporting Information).^[^ ^26^, ^30^ ^]^ The consistent ^1^H NMR spectra and mass spectra of these molecules with those in previous reports indicate their successful preparation (Figures S1–S12). The optical properties of these D–A structured molecules were investigated by UV–vis–NIR spectroscopy. These molecules exhibit strong absorption in the vis‐to‐NIR range in dichloromethane (DCM) (**Figure** **2**a). **DMA‐TCNQ** shows the lowest‐energy absorption band with *λ*_abs_ centered at 767 nm and molar absorption coefficient (*ε*) of 1.14 × 10^4^ M^−1^ cm^−1^, while **DPA‐TCNQ**, **DMA‐TCNE**, and **DPA‐TCNE** show theirs with *λ*_abs_ centered at 757 nm (*ε* = 0.62 × 10^4^ M^−1^ cm^−1^), 571 nm (*ε* = 0.60 × 10^4^ M^−1^ cm^−1^), and 600 nm (*ε* = 0.61 × 10^4^ M^−1^ cm^−1^), respectively. It is worth noting that the *λ*_abs_ do not shift apparently by changing the D units (i.e., shifted by 172.2 cm^‒1^ from **DPA‐TCNE** to **DMA‐TCNE**) whereas significant absorption red‐shift is observed by replacing the A units (i.e., shifted by 4475.3 cm^‒1^ from **DMA‐TCNE** to **DMA‐TCNQ**), which is attributed to the strengthened D–A conjugation after introducing the strongly electron‐withdrawing TCNQ.

The absorption profiles of these D–A structured molecules in 1% DMSO PBS solution were collected to preliminarily evaluate the appropriateness of these molecules as PTAs in living organisms. Expectedly, absorption profiles of these molecules follow the same trend as in DCM, apparently keeping a consistent nature albeit with more resolved lowest‐energy bands due to the preferential contribution of constituent transitions (Figure 2b). Due to the low solubility of these D–A structured molecules in water, they tend to self‐assemble to form NP aggregates through intermolecular interaction such as *π*–*π* stacking and *p*‐*π* interaction, as demonstrated in their single‐crystal structures. The transmission electron microscope (TEM) images showed that all these molecules are spherical in morphology (Figure S16, Supporting Information). The size distribution of these molecules in 1% DMSO PBS solution was collected through dynamic light scattering (DLS) measurement. The particle sizes were found to distribute in a range of 100–200 nm (Figures 2c–f), suitable for tissue penetration, effective light absorption, and subsequent light‐to‐heat generation during the PTT process.

### 2.3 Photothermal Conversion Investigation

To investigate their photothermal activities, photothermal conversion experiments were conducted on D–A structured molecules in 1% DMSO PBS solution using a 760‐nm LED lamp as the light source and infrared thermal camera for real‐time recording of temperature changes (Δ*T*). Upon photoirradiation using a 760‐nm LED lamp for 10 min, the photothermal conversion performance of all studied molecules grew as time elapsed, along with increasing concentration of the molecules studied as well as power densities of the photoirradiation source, with 80 µm **DMA‐TCNQ** at 1.2 W cm^−2^ showing the best performance (Figures S17 and S18, Supporting Information). Considering the highest Δ*T* in photothermal conversion, DMA works better than DPA as a D unit while TCNQ is a more efficient A unit than TCNE, as reflected by the trend in Δ*T* (**DMA‐** **TCNQ**, 30.65 °C > **DPA‐TCNQ**, 22.08 °C > **DMA‐TCNE**, 9.45 °C > **DPA‐TCNE**, 8.02 °C) (Figure 2g; Figure S19, Supporting Information). Provided that Δ*T* by the 1% DMSO PBS solution is 4.19 °C, the designed D–A structured molecules are indeed active PTAs. Based on the result of the heating–cooling experiment, PCEs of **DMA‐TCNE**, **DPA‐TCNE**, **DMA‐TCNQ**, and **DPA‐TCNQ** were calculated as 88.2, 78.9, 91.3 and 79.9% (Figure 2h; Table S7, Supporting Information).^[^ ^31^ ^]^ To the best of our knowledge, the **DMA‐TCNQ** has the highest PCE value among all reported molecular PTA until now (Table S8, Supporting Information). Also, all D–A structured molecules are photothermally stable as revealed by steady photothermal conversion performance (with a slight decline of 1.88–6.78%) upon 5 cycles of operation (Figure 2i) together with unchanged UV–vis–NIR spectra after the photothermal process (Figure S20, Supporting Information). The highest Δ*T* and the best PCEs of **DMA‐TCNQ** may arise from its strongest light harvesting capacity making it the most suitable candidate for the following PTT study.

**Figure 3:**
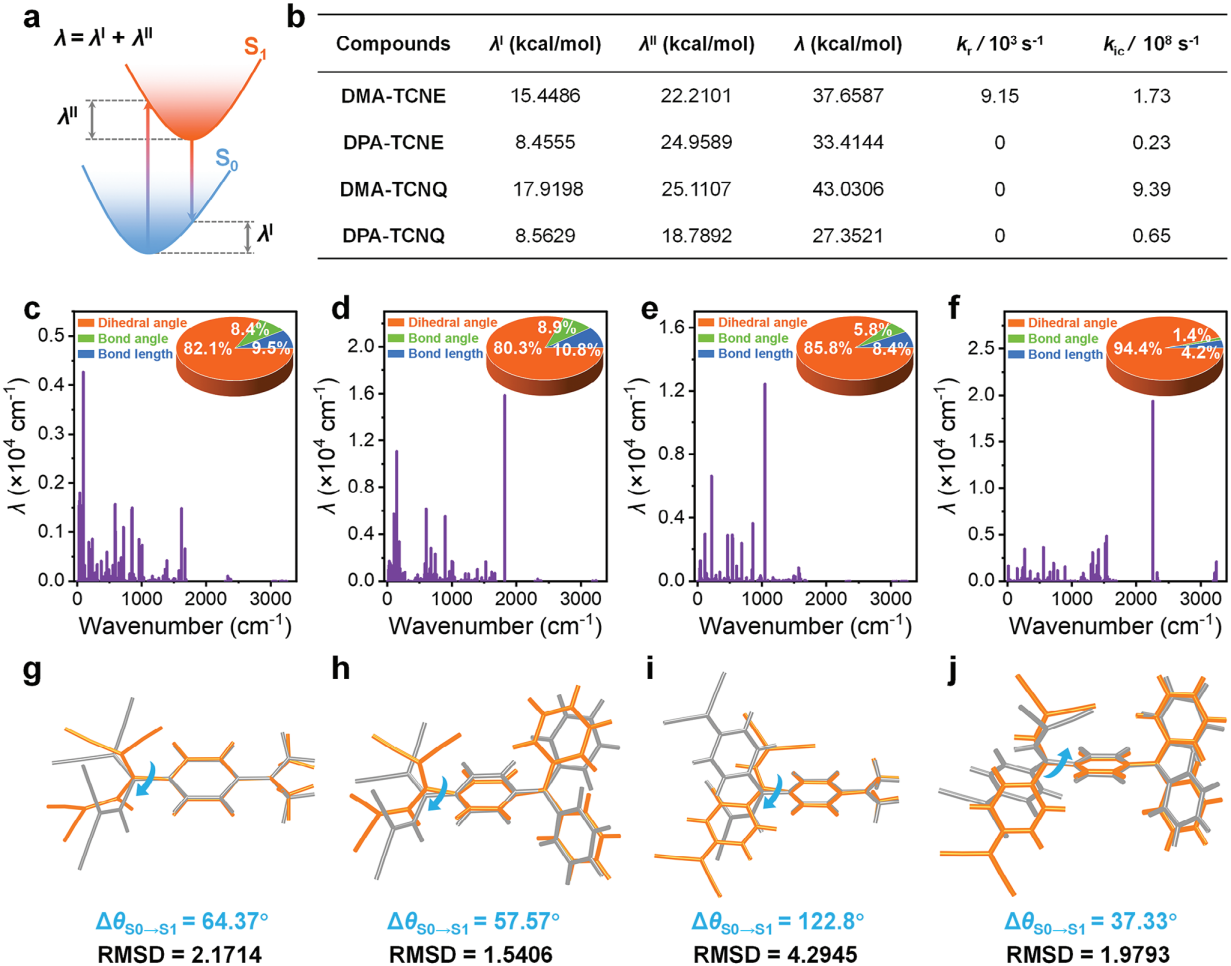
a) Schematic representation of the reorganization energy for four‐point calculations, b) summary of reorganization energy, radiative transition rate, and nonradiative transition rate for D–A structured molecules through the theoretical calculations, calculated total reorganization energy versus normal mode frequencies for c) **DMA‐TCNE**, d) **DPA‐TCNE**, e) **DMA‐TCNQ**, and f) **DPA‐TCNQ**, insets are contributions to the total reorganization energy from dihedral angle, bond length, and bond angle, minimum energy geometries calculated for the S_0_ (gray) and S_1_ (orange) for g) **DMA‐TCNE**, h) **DPA‐TCNE**, i) **DMA‐TCNQ**, and j) **DPA‐TCNQ**.

To rationalize the photothermal conversion performance of the D–A structured molecules, theoretical calculation was employed to estimate the energy‐consuming deactivation from the excited state to the ground state. The reorganization energies (*λ*) of the D–A structured molecules were calculated through four‐point calculations (**Figure** **3**a). They all have large total *λ* values in the range of 27.3521–43.0306 kcal mol^−1^ for **DMA‐TCNQ**, **DPA‐TCNE**, **DMA‐TCNQ**, and **DPA‐TCNQ** (Figure 3b). As shown in Figures 3c–f, the D–A structured molecules exhibit low‐frequency vibration, principally contributed by molecular motion between dihedral planes. Besides, all these molecules show large internal conversion rate constants (*k*_IC_) (0.23 ‒ 9.39 × 10^8^ s^‒1^) with **DMA‐TCNQ** holding the largest value. On the other hand, only **DMA‐TCNE** possesses a non‐zero radiative decay rate constant (*k*_r_). These results rationalize the best photothermal conversion capacity of **DMA‐TCNQ**. Considering the optimized structures of these molecules, they all have significant differences between the ground state (S_0_) and excited state (S_1_). Importantly, the **DMA‐TCNQ** shows the largest root mean squared displacement (RMSD) of 4.2945 Å and the largest extent of twisting between D and A units (dihedral angles of 122.8°) indicative of non‐radiative deactivation of excited state via rapid intramolecular vibrational/rotational motions (Figures 3g–j). These results echo our design concept and reveal the structure‐function correlation of these D–A structured molecules.

### 2.4 Photothermal Cytotoxicity in Cells

**Figure 4:**
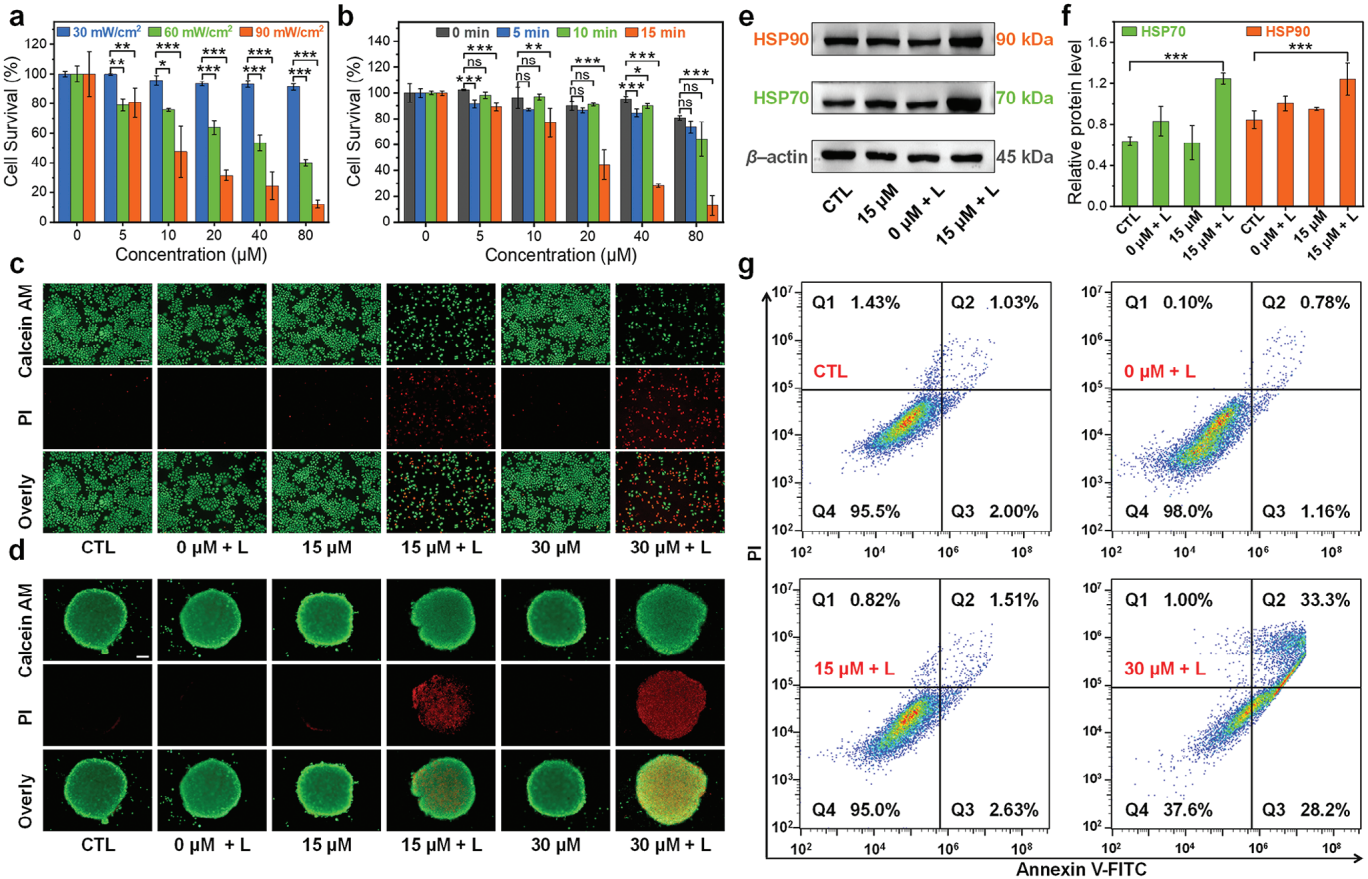
Cell viability of NCl‐H460 cells with different concentrations of **DMA‐TCNQ**. a) After 0.5 h of incubation, cells were irradiated with a 760‐nm LED lamp (30, 60, and 90 mW cm^−2^) irradiation for 20 min, b) after 0.5 h of incubation, cells were irradiated for different time durations (0, 5, 10, and 15 min) with a 760‐nm LED lamp (90 mW cm^−2^), Error bars, mean ± SD (*n* = 4). * *p* < 0.05, ** *p* < 0.01, *** *p* < 0.001, c,d) fluorescence images of living and dead NCI‐H460 cells (in both 2D monolayers and 3D tumorspheres) with calcein AM/PI staining and after different treatments, scale bar = 100 µm, e–f) western blot analysis and quantification of HSP70 and HSP90 proteins after a 24‐h treatment (separated into four groups, including “control”, “0 µm + L”, “15 µm + L”, and “30 µm + L”, Light: 760‐nm LED lamp, 90 mW cm^−2^, 20 min) Error bars, mean ± SD (*n* = 3), * *p* < 0.05, ** *p* < 0.01, *** *p* < 0.001, g) flow cytometry analysis of NCI‐H460 cells using Annexin V‐FITC and PI staining following different treatments (cells were incubated under various conditions and separated into four groups, including “control”, “0 µm + L”, “15 µm + L”, and “30 µm + L”).

The intense broad absorption, high PCE and appropriate particle size jointly highlight **DMA‐TCNQ** as the most promising candidate in this pool of D–A structured molecules for PTT. Lung cancer remains one of the leading causes of global mortality, with ≈1.8 million deaths expected by 2020. Especially, non‐small cell lung cancer does not respond to some chemotherapy drugs, accounting for 85% of lung cancer cases, with a 5‐year survival rate of < 15%.^[^ ^32^ ^]^ Therefore, it is crucial to search for more effective treatment for non‐small cell lung cancer. Before moving to the in vivo stage, the in vitro cytotoxicity of **DMA‐TCNQ** to tumor cells was evaluated by 3‐[4,5‐dimethylthiazol‐2‐yl]−2,5‐diphenyltetrazolium bromide (MTT) assay 24 h after treatments. Specifically, the treatment was executed by incubating human non‐small cell lung carcinoma (NCI‐H460 cell line) with varying concentrations of **DMA‐TCNQ** for 30 min followed by subsequent irradiation using a 760‐nm LED lamp at different power densities for a duration of 20 min or at 90 mW cm^−2^ for various time durations (along with a control group in dark). For the incubation group without irradiation, cell survival reached over 80% (**Figures** **4**a,b), indicative of low cytotoxicity of **DMA‐TCNQ** at dark. On the contrary, cell viability fell along with a higher concentration of **DMA‐TCNQ**, larger power intensity of irradiation source, and longer irradiation duration with the lowest cell viability of 10% given by 80 µm of **DMA‐TCNQ** irradiated at 90 mW cm^−2^ for 20 min, signifying the importance of light intensity, irradiation duration and concentration of **DMA‐TCNQ** in photoinduced cytotoxicity toward NCI‐H460 cells. Hemolysis tests are often used to check the blood compatibility of materials. As shown in Figure S21 (Supporting Information), the hemolysis rate is below the safety standard of 5%, indicating good hemocompatibility of **DMA‐TCNQ** in blood circulation. Next, the PTT anticancer capacity of **DMA‐TCNQ** was studied via fluorescence imaging with living/dead cells stained by calcein‐AM (green fluorescence)/propidium iodide (PI, red fluorescence) for visual differentiation. As displayed in Figures 4c,d, only green fluorescence was observed for the groups either under irradiation in the absence of **DMA‐TCNQ** “0 µm + L” or added **DMA‐TCNQ** without irradiation “15 µm” and “30 µm”. In sharp contrast, strong red fluorescence was noticed in the group “**DMA‐TCNQ** + L” with both **DMA‐TCNQ** added and irradiation. To note, considering the 3D tumor sphere of the “30 µm + L” group cells were found not clustering (Figure 4d). These results indicate that PTT treatment can kill NCI‐H460 cells in both 2D monolayers and 3D tumorspheres, echoing the results in plots depicted in Figures 4a–d. In addition, through the results of western blot experiments (Figures 4e,f), it was showed that the levels of heat shock proteins HSP70 and HSP90 were slightly elevated in the PTT probably stemming from upregulation by heat shock proteins to prevent damage of cell structure upon thermal stimulation.^[^ ^33^ ^]^ Meanwhile, effective cell apoptosis was executed by incubating cells with **DMA‐TCNQ** followed by subsequent irradiation using a 760‐nm LED lamp by means of flow cytometry analysis (Figure 4g).

### 2.5 Photothermal Therapy In Vivo

**Figure 5:**
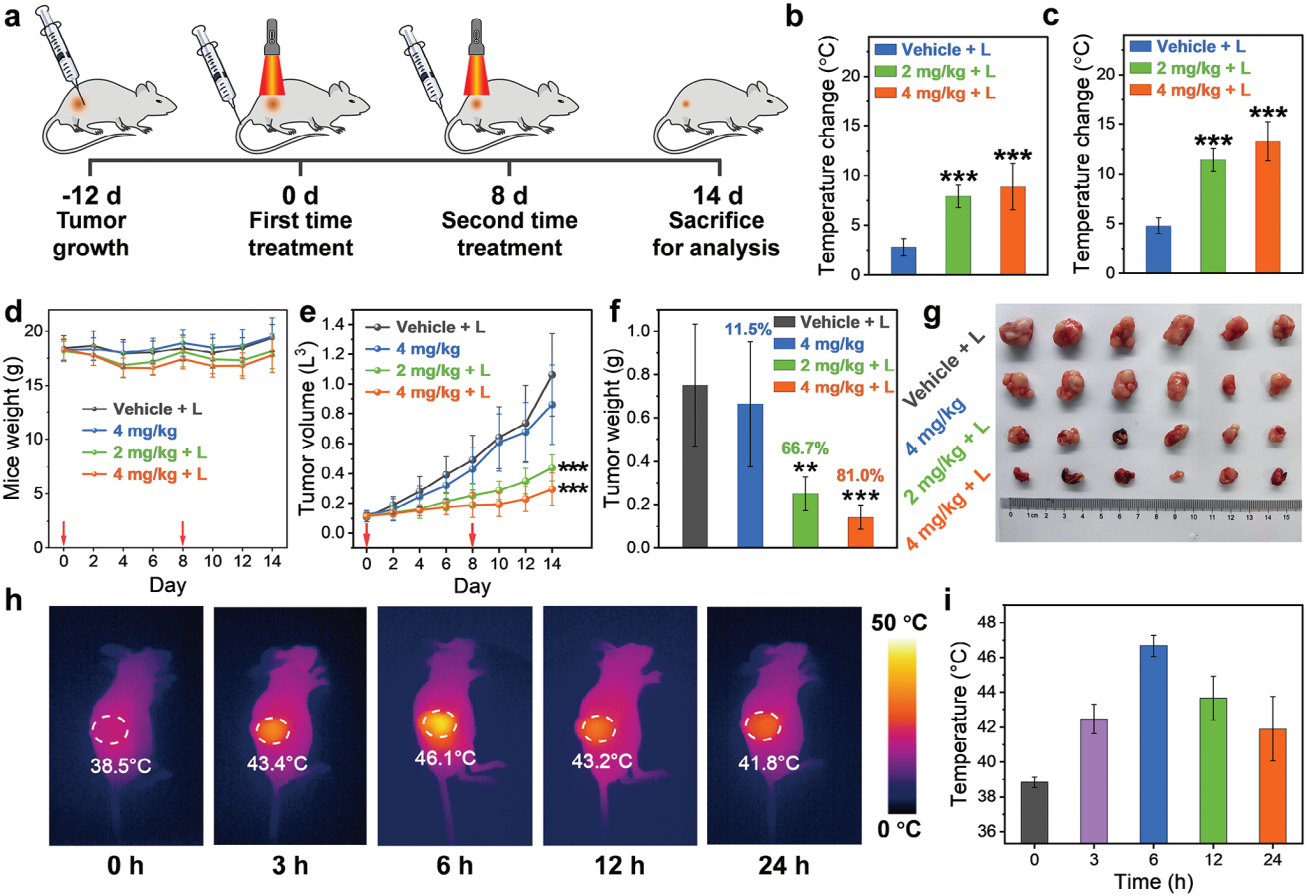
a) Schematic illustration of the PTT treatment, b, c) temperature changes at the tumor site irradiated with a 760‐nm LED lamp (1.2 W cm^−2^) for 10 min from infrared images of nude mice on (b) day 0 and (c) day 8, d) body weight curves, e) tumor volume curves, f) final tumor weight of mice, g) photograph of final tumor nodules of the vehicle group and the photothermal therapy of **DMA‐TCNQ** groups. Error bars, mean ± SD (*n* = 6), statistical significance was determined at * *p* < 0.05, ** *p* < 0.01, *** *p* < 0.001, representing significant difference compared with the “Vehicle + L”), red arrows indicate time points of intravenous injection treatments, h) IR images of NCI‐H460 lung tumor‐bearing BALB/c nude mice at different time after intravenous injection of **DMA‐TCNQ** (irradiation by 760‐nm LED lamp, 4 mg kg^−1^ **DMA‐TCNQ**), i) The final temperature at the tumor site after 10 min of light exposure. Error bars, mean ± SD (*n* = 3).

Driven by the in vitro results, the in vivo PTT of **DMA‐TCNQ** was then evaluated. As illustrated in **Figure** **5**a, the tumor was subcutaneously grown by inoculating 0.1 mL RPMI solution of 3 × 10^6^ NCI‐H460 cells on the auxiliary region of female BALB/c nude mice and cultivated for 12 days before the treatment started. The tumor‐bearing mice were randomly divided into four groups (*n* = 6), designated as “Vehicle + L”, “4 mg kg^−1^”, “2 mg kg^−1^ + L”, and “4 mg kg^−1^ + L”. These groups of mice were intravenously injected with 0.9% NaCl solution containing 10% PET with/without **DMA‐TCNQ**. Tumors were irradiated for 10 min with a 760‐nm LED lamp (1.2 W cm^−2^) 6 h after intravenous injection. The temperatures before and after treatment were recorded using a thermal imaging camera. The second treatment was conducted on day 8 and the mice were sacrificed for analysis at day 14. The tumor volume and mouse weight were monitored every 2 days throughout the treatment, and the tumors were extracted and throughout the treatment, and the tumors were extracted and weighed at the end of the experiment. The in vivo antitumoral effect of the different treatments was evaluated by examining tumor volume change over a 14‐day duration (starting from the first day of treatment) and the ultimate tumor weight at the end of the treatment.

The results revealed that **DMA‐TCNQ** can effectively raise the temperature in intra‐tumoral regions upon irradiation by a 760‐nm LED lamp during the PTT period. Looking into the first treatment, relatively small temperature changes (Δ*T* = 2.8 °C) in tumors were observed from the “Vehicle + L” group, while rapid temperature increases in tumors were observed from the “2 mg kg^−1^ + L” (Δ*T* = 7.9 °C), and “4 mg kg^−1^ + L” groups (Δ*T* = 8.9 °C) (Figure 5b). It is noted that before 760‐nm LED lamp irradiation, there was an even distribution of IR heat signals in nude mice without any noticeable temperature elevations (Figure S22; Figure S23, Supporting Information). However, after 760‐nm LED lamp irradiation on the tumor sites, distinct temperature rises, exceeding 46.4 °C, were observed. During the subsequent treatment, tumors in the “Vehicle + L” group showed a temperature elevation by 4.8 °C, while the “2 mg kg^−1^ + L” and “4 mg kg^−1^ + L” groups exhibited more significant temperature increase in the tumor region, reaching a higher temperature of 49.6 °C (Δ*T* = 11.4 °C) for “2 mg kg^−1^ + L” groups and 50.7 °C (Δ*T* = 13.3 °C) for “4 mg kg^−1^ + L” groups (Figure 5c). Noteworthily, the nude mice exhibited no signs of discomfort, maintained good growth status, normal feeding, and excretion, and only experienced partial crusting at the tumor site throughout the treatment period (Figure S24, Supporting Information).

As depicted in Figure 5d constant mice body weights of all four groups over the whole treatment period suggest that tail vein treatment exerts little impact on the growth of mice. Furthermore, normal growth of the tumor in the “Vehicle + L” group indicates no photo‐driven cytotoxicity in the absence of **DMA‐TCNQ** injection (Figure 5e). It is worth noting that the “4 mg kg^−1^” groups only showed limited tumor suppression, suggesting the insignificant dark cytotoxicity of **DMA‐TCNQ**. In stark contrast, a substantial reduction in tumor weight and tumor volume can be observed in both “2 mg kg^−1^ + L” and “4 mg kg^−1^ + L” groups when compared with the control group (Figure 5f,g). Specifically, the tumor of the control group was heavier than “2 mg kg^−1^ + L” and “4 mg kg^−1^ + L” by 3.0 and 5.2 times, respectively, indicating statistical significance and demonstrating the effectiveness of the intravenous injection treatment. Figure 5g clearly shows better tumor suppression in the treatment group than in the control group. Especially, in the “4 mg kg^−1^ + L” group, the degree of vacuolization in tumors was considerably enhanced, and tumor cells were ablated more effectively. The apparent effect of the drugs can be rationalized by the enrichment of PTA in the tumor site possibly via enhanced permeation and retention (EPR) effect, even distribution on the tumor surface and penetration into the interior of the tumor. In addition, photothermal imaging was employed to examine tumor accumulation and metabolism. **DMA‐TCNQ** was intravenously injected into mice, followed by photothermal imaging (PTI) in vivo. After 6 h of injection, the photothermal signal intensity reached its peak (46.1 °C) at the tumor site (Figure 5h), indicating the maximum accumulation of **DMA‐TCNQ**. The temperature decreased to 41.8 °C after 24 h (Figure 5i). These findings indicate that **DMA‐TCNQ** was effectively enriched at the tumor site 6 h after injection, followed by metabolic clearance starting 12 h later. Furthermore, hematoxylin and eosin (H&E) staining results of “4 mg kg^−1^ + L” group showed a significant decrease in the number of cancer cells in tumor samples, while no significant damage was observed in other organ sections, indicating that PTT caused damage to tumor tissue and had no toxicity to other organs (Figure S25, Supporting Information). As a whole, the in vivo findings highlight the significant antitumor activity of **DMA‐TCNQ** by intravenous injection phototherapy.

## 3 Conclusion

Based on the selection of D–A junction, four D–A structured twisted molecules (**DMA‐TCNE**, **DPA‐TCNE**, **DMA‐TCNQ**, and **DPA‐TCNQ**) were designed and prepared. Importantly, the interplanar dihedral angles between the D and A units play a critical role in the light‐harvesting nature and PCEs of these molecules. Out of these molecules, **DMA‐TCNQ** was found to exhibit good photostability and high photothermal conversion ability (*η* = 91.3%), lower dark toxicity as well as effective NIR phototoxicity and inhibit the growth of tumors in vivo significantly, providing the possibility for further antitumor therapy pinpointing larger or deeper buried tumors. This work undoubtedly sheds light on the development of PTAs for PTT by rational structural design and through facile *CA–RE* reaction, thereby broadening the scope of potential PTAs.

## 4 Experimental Section

### Preparation of Nanoparticles

A certain concentration of D–A structured molecules (e.g., 1.0 mm, 0.1 mL) was dissolved in DMSO and then diluted into 10.0 mL PBS solution under sonication. In order to obtain the target nanoparticles, a further 45 min of sonication was required.

### Calculation of the Photothermal Conversion Efficiency

According to the previous method, the conversion efficiency was determined. As an example, the **DMA‐TCNQ** is displayed as follows:^[^ ^31^ ^]^

Based on the total energy balance for this system:

(1) $$\sum_{i}m_{i}C_{p,i}\frac{dT}{dt}=Q_{s}-Q_{\mathrm{loss}}$$

where *m*_i_ (1.5 g) and *C*_p,i_ (4.18 J g^−1^ °C^−1^) are the mass and heat capacity of system components, respectively. *Q*_s_ is the photothermal heat energy inputted by a 760‐nm LED lamp to the sample solution, and *Q*_loss_ is thermal energy lost to the surroundings. When the temperature is maximum, the system is in balance:

(2) $$Q_{s}=Q_{\mathrm{loss}}=hS\Delta T_{\max}$$

where *h* is the heat transfer coefficient, *S* is the surface area of the container, Δ*T*_max_ is the maximum temperature change. The photothermal conversion efficiency *η* is calculated from the following equation:

(3) $$\eta =\frac{hS\Delta T_{\max}}{I\left(1-10^{-A_{760}}\right)}$$

where *I* is the laser power (1.2 W cm^−2^) and *A*_760_ is the absorbance of the samples at the wavelength of 760 nm (0.54295 for **DMA‐TCNQ**).

In order to obtain the *hS*, a dimensionless driving force temperature, *θ* is introduced as follows:

(4) $$\theta =\frac{T-T_{\mathrm{surr}}}{T_{\max}-T_{\mathrm{surr}}}$$

where *T* is the temperature of the solution, *T*_max_ is the maximum system temperature (59.09 °C for **DMA‐TCNQ**), and *T*_surr_ is the initial temperature (28.44 °C).

The sample system time constant *τ*_s_ is obtained as follows:

(5) $$\tau_{s}=\frac{\sum_{i}m_{i}C_{p,\;i}}{hS}$$

thus

(6) $$\frac{d\theta }{dt}=\frac{1}{\tau_{s}}\;\frac{Q_{s}}{hS\Delta T_{\max}}-\frac{\theta }{\tau_{s}}$$

when the laser is off,

(7) $$Q_{s}=0\text{, therefore}\;\frac{d\theta }{dt}=-\frac{\theta }{\tau_{s}},\;\;\;\mathrm{and}\;\;\;t=-\tau_{s}\ln\theta$$

so *hS* could be calculated from the slope of cooling time versus −ln*θ*. Therefore, *τ*_s_ is 245.9 s, and the photothermal conversion efficiency *η* is 91.3%.

### Photothermal Effect of PTT on Cells

In the typical MTT assay, NCI‐H460 cells were seeded in 96‐well plates at a density of 5000 cells per well. After cells were adherent, different concentrations of **DMA‐TCNQ** were added for 30 min incubation and then irradiated with a 760‐nm LED at different power for 20 min or at 90 mW cm^−2^ for different times. 24 h later, MTT was added and the cells were incubated for another 2 h. The absorbance of each well was read by an enzyme marker (TECAN, Switzerland) at 570 nm.

### Calcein‐AM/PI Staining in 2D Monolayer Cells

The experiment was performed according to the protocol described in the Calcein/PI Live/Dead Assay Kit (Beyotime, China). Briefly, cells were seeded in 12‐well plates. Cells were treated with **DMA‐TCNQ** and incubated for 30 min, then irradiated with a 760‐nm LED lamp at 90 mW cm^−2^ for 20 min. After 24 h, cells were rinsed twice with PBS and then incubated with Calcein‐AM/PI (1000X) for 30 min at 37 °C in the dark. The results of Calcein‐AM/PI staining were observed through an inverted fluorescence microscopy (Axio Observer A1, Germany).

### Hemolysis Assays of **DMA‐TCNQ**

The hemolytic ability of the **DMA‐TCNQ** was evaluated using rabbit red blood cells (rRBCs) according to a previously reported method.^[^ ^34^ ^]^ The red blood cells were pelleted by centrifugation (1000 × *g*, 10 min), washed three times with phosphate‐buffered saline (PBS), and resuspended in PBS to obtain a 5% v/v suspension. Subsequently, 500 µL of the rRBC suspension was mixed with an equal volume of sample solution at different concentrations and incubated at 37 °C for 24 h. The mixture was centrifuged at 1000 × *g* for 10 min, and then 30 µL of the supernatant was diluted with 100 µL of PBS in a 96‐well plate. PBS and 1% Triton X‐100 treatments served as negative and positive controls, respectively. The OD value was measured at 540 nm using a microplate reader (INFINITE 200 PRO M Nan, Switzerland).

Calculation of the hemolysis rate:

(8) $$\begin{array}{ccc} \mathrm{Hemolysis}\;\left(\%\right) & = & \left[\left(OD_{\mathrm{Sample}}-OD_{\mathrm{PBS}}\right)/\left(OD_{\mathrm{Triton}\;X-100}-OD_{\mathrm{PBS}}\right)\right]\\ & & \times 100\% \end{array}$$

A total of 4% rRBCs were purchased from Guangzhou Humayun Biotech Co., LTD.

### Calcein‐AM/PI Staining in Multicellular Tumor Spheroid Model

Heat the suspension of 0.75% agarose in PBS buffer in a high‐pressure sterilizer. Transfer the hot lotion into the holes of 96 cells culture well plate (40 µL per well). Expose the culture dish to ultraviolet radiation for 3 h to ensure sterility and allow the agarose solution to cool. Afterward, the agarose was covered with NCI‐H460 cell suspension at a density of 5000 cells per well in 100 µL of medium. MCTS was incubated and maintained in a cell culture incubator at 37 °C with 5% CO_2_. The cell suspension formed MCTS after 5 days. Then medium containing 15 or 30 µm **DMA‐TCNQ** was added to the experimental group and co‐cultivated for 30 min followed by 20 min of light exposure at 90 mW cm^−2^. After 24 h, cells were rinsed twice with PBS and then incubated with Calcein‐AM/PI (1000X) for 30 min at 37 °C in the dark. The results of Calcein‐AM/PI staining were observed through an inverted fluorescence microscopy (Axio Observer A1, Germany).

### Cell Apoptosis Assay

NCI‐H460 cells were seeded into 6‐well plates. Cells were treated with **DMA‐TCNQ** and incubated for 30 min, then irradiated with 760‐nm LED lamp of 90 mW cm^−2^ for 20 min. After 24 h, the cells were collected, centrifuged at 1000 rpm for 5 min, washed with PBS twice, resuspended in 500 µL binding buffer (1×), and stained with 5 µL Annexin V and 5 µL propidium iodide for 10 min at room temperature. The results of the Annexin V‐FITC/PI apoptosis assay were obtained from flow cytometry (Accuri c6, BD, USA).

### Western Blot

NCI‐H460 cells were seeded in 6‐well plates with a density of 500 000 per well. After 12 h, fresh medium containing **DMA‐TCNQ** (15 µm) was added for 30 min incubation, and then the cells were irradiated by a 760‐nm LED lamp for 20 min. After 24 h, the protein was harvested and quantified using the BCA protein assay kit. Then, the protein sample was separated on 10% SDS‐PAGE and transferred to a nitrocellulose membrane (BOSTER). Subsequently, the membranes were closed with 5% skimmed milk for 1 h at room temperature and probed with primary antibody overnight at 4 °C and incubated with secondary antibody (anti‐rabbit or anti‐mouse, 1:10 000) for 1 h at room temperature. Target proteins were detected using High ChemiDoc XRS (Bio‐Rad ChemiDocXRS+, USA).

### Animal Test Articles

To prepare the stock solution of the test articles, the **DMA‐TCNQ** was dissolved by PET and administered within 30 min. Prior to administration, working solutions were prepared by diluting the stock solution with a Stroke‐physiological saline solution (NS), with the final concentration of PET being 10% v/v. PET solution = (polyethylene glycol 400, 60%; ethanol 30%; Tween 80, 10%).

### Cells and Animals

Human Lung Carcinoma NCI‐H460 cells were from the ATCC collection. Female BALB/c SPF nude mice weighing 14–16 g were supplied by Guangdong Vital River Laboratory Animal Technology Co., LTD, with an approval code of SCXK(粤)2022‐0063.

### Housing

The animals were housed in an SPF‐grade animal laboratory that met the requirements of the SPF grade for animal testing facilities. The temperature was maintained within the range of 22–26 °C and the humidity was kept between 40–70%. The approval number of the SPF animal laboratory was SYXK (粤)2022‐0079.

### Accumulation and Metabolism of **DMA‐TCNQ** in Tumor

Benefiting from the excellent photothermal conversion efficiency of **DMA‐TCNQ** (91.3%), photothermal imaging (PTI) was used to study the accumulation and metabolism of **DMA‐TCNQ** at the tumor site after intravenous injection. For in vivo photothermal imaging at 3, 6, 12, and 24 h after intravenous administration with **DMA‐TCNQ** (4 mg kg^−1^, prepared as before), the infrared thermal images of mice were acquired using an IR camera after the irradiation of 760‐nm LED lamp (1.2 W cm^−2^) for 10 min. The photothermal image at 0 h was acquired before the injection of **DMA‐TCNQ**.

### Inoculation

A total of 3 × 10^6^ NCI‐H460 cells were suspended in 0.1 mL free‐FBS RPMI culture medium and subcutaneously inoculated into the right back flank region of female BALB/c nude mice.

### Statistical Analysis of Tumor Volume

The length (*L*, mm) of the longest tumor axis and the length (*W*, mm) of the axis vertical to the longest axis (width) were measured with a slide caliper, to obtain the tumor volume (*V*, mm^3^), which was calculated using the following Equation.

(9) $$V=\frac{L\times W^{2}}{2}$$

### Statistical Analysis of Tumor Growth Inhibition

Taking the mean tumor weight of the model control and treatment groups at the end of the experiment as G(C) and G(T), respectively, tumor growth inhibition was calculated using the following Equation.

(10) $$\mathrm{Tumor}\;\mathrm{growth}\;\mathrm{inhibition}\;\left(\%\right)=\left(1-\frac{G\left(T\right)}{G\left(C\right)}\right)\times 100$$

All data was expressed as Mean ± SD. Comparisons between groups were tested by SPSS 20.0 One‐Way ANOVA analysis and the Least Significant Difference (LSD) test.

### In Vivo Biocompatibility Evaluation

To investigate the systemic toxicity of various treatments, the tumor tissues and main organs (heart, liver, spleen, lung, and kidney) of mice from Blank, 4 mg kg^−1^ and 4 mg kg^−1^ + L were collected. Afterward, all the collected tissues were fixed with 4% paraformaldehyde for hematoxylin and eosin (H&E) staining.

## Conflict of Interest

The authors declare no conflict of interest.

## Supporting information

Supporting Information

## Data Availability

The data that support the findings of this study are available from the corresponding author upon reasonable request.

## References

[1] F. Zhao , W. Huang , L. He , S. Nie , Z. Sun , T. Chen , H. Yin , J. Zhao , Nano Today 2023, 50, 101819.

[2] a) L. Galluzzi , J. M. Bravo‐San Pedro , S. Demaria , S. C. Formenti , G. Kroemer , Nat. Rev. Clin. Oncol. 2017, 14, 247; 27845767 10.1038/nrclinonc.2016.183

[3] a) Z. Deng , H. Li , S. Chen , N. Wang , G. Liu , D. Liu , W. Ou , F. Xu , X. Wang , D. Lei , P.‐C. Lo , Y. Y. Li , J. Lu , M. Yang , M.‐L. He , G. Zhu , Nat. Chem. 2023, 15, 930; 37353602 10.1038/s41557-023-01242-w

[4] a) S. Kuang , F. Wei , J. Karges , L. Ke , K. Xiong , X. Liao , G. Gasser , L. Ji , H. Chao , J. Am. Chem. Soc. 2022, 144, 4091; 35171598 10.1021/jacs.1c13137

[5] a) W. Huang , L. He , Z. Zhang , S. Shi , T. Chen , ACS Nano 2021, 15, 20225; 34807558 10.1021/acsnano.1c08237

[6] a) H. S. Jung , P. Verwilst , A. Sharma , J. Shin , J. L. Sessler , J. S. Kim , Chem. Soc. Rev. 2018, 47, 2280; 29528360 10.1039/c7cs00522a PMC5882556

[7] a) N. Fernandes , C. F. Rodrigues , A. F. Moreira , I. J. Correia , Biomater. Sci. 2020, 8, 2990; 32355937 10.1039/d0bm00222d

[8] Z. Xu , Y. Zhang , W. Zhou , L. Wang , G. Xu , M. Ma , F. Liu , Z. Wang , Y. Wang , T. Kong , B. Zhao , W. Wu , C. Yang , J. Nanobiotechnol. 2021, 19, 137. 10.1186/s12951-021-00884-7 PMC8120736 33985525

[9] D. An , J. Fu , B. Zhang , N. Xie , G. Nie , H. Ågren , M. Qiu , H. Zhang , Adv. Funct. Mater. 2021, 31, 2101625.

[10] J. Li , K. Pu , Chem. Soc. Rev. 2019, 48, 38. 30387803 10.1039/c8cs00001h

[11] a) H. Li , Y. Kim , H. Jung , J. Y. Hyun , I. Shin , Chem. Soc. Rev. 2022, 51, 8957; 36226744 10.1039/d2cs00722c

[12] Z. Xia , N. Xue , W. Shi , C. Lu , J. Phys. Chem. C 2019, 123, 30536.

[13] a) Z. He , L. Zhao , Q. Zhang , M. Chang , C. Li , H. Zhang , Y. Lu , Y. Chen , Adv. Funct. Mater. 2020, 30, 1910301;

[14] a) D. Escudero , Acc. Chem. Res. 2016, 49, 1816; 27575871 10.1021/acs.accounts.6b00299

[15] Y. Cai , Z. Wei , C. Song , C. Tang , X. Huang , Q. Hu , X. Dong , W. Han , Chem. Commun. 2019, 55, 8967. 10.1039/c9cc04195h 31290491

[16] P. Liang , Y. Wang , P. Wang , J. Zou , H. Xu , Y. Zhang , W. Si , X. Dong , Nanoscale 2017, 9, 18890. 29177329 10.1039/c7nr07204j

[17] Y. Cai , P. Liang , Q. Tang , X. Yang , W. Si , W. Huang , Q. Zhang , X. Dong , ACS Nano 2017, 11, 1054. 28033465 10.1021/acsnano.6b07927

[18] Y. Cai , P. Liang , W. Si , B. Zhao , J. Shao , W. Huang , Y. Zhang , Q. Zhang , X. Dong , Org. Chem. Front. 2018, 5, 98.

[19] a) Z. Jiang , C. Zhang , X. Wang , M. Yan , Z. Ling , Y. Chen , Z. Liu , Angew. Chem., Int. Ed. 2021, 60, 22376; 10.1002/anie.202107836 34289230

[20] a) L. Feng , C. Li , L. Liu , X. Chen , G. Jiang , J. Wang , B. Z. Tang , Angew. Chem., Int. Ed. 2022, 61, e202212673; 10.1002/anie.202212673 36256574

[21] a) Y. Li , K. Ding , H. Wu , Q. Wan , Y. Ma , Y. Huang , Z. Wang , W. Zhang , J. Hou , B. Z. Tang , Mater. Chem. Front. 2022, 6, 316;

[22] a) W. Jiang , Z. Zhang , M. Ye , S. Pan , G. Huang , T. Chen , X. Zhu , Nano Today 2022, 46, 101598;

[23] T. Michinobu , F. Diederich , Angew. Chem., Int. Ed. 2018, 57, 3552. 10.1002/anie.201711605 29469183

[24] X. Tang , Z. Chen , Q. Xu , Y. Su , H. Xu , S. Horike , H. Zhang , Y. Li , C. Gu , CCS Chem 2022, 4, 2842.

[25] Z. Lin , Y.‐H. Zhong , L. Zhong , X. Ye , L.‐H. Chung , X. Hu , Z. Xu , L. Yu , J. He , JACS Au 2023, 3, 1711. 37388679 10.1021/jacsau.3c00132 PMC10302748

[26] a) C. Philippe , A. T. Bui , S. Batsongo‐Boulingui , Z. Pokladek , K. Matczyszyn , O. Mongin , L. Lemiègre , F. Paul , T. A. Hamlin , Y. Trolez , Org. Lett. 2021, 23, 2007; 33635667 10.1021/acs.orglett.1c00136 PMC8155560

[27] a) T. Lu , F. Chen , J. Comput. Chem. 2012, 33, 580; 22162017 10.1002/jcc.22885

[28] a) S. Chen , N. Ullah , R. Zhang , J. Phys. Chem. Lett 2018, 9, 4857; 30085672 10.1021/acs.jpclett.8b01972

[29] a) X. Tang , L.‐S. Cui , H.‐C. Li , A. J. Gillett , F. Auras , Y.‐K. Qu , C. Zhong , S. T. E. Jones , Z.‐Q. Jiang , R. H. Friend , L.‐S. Liao , Nat. Mater. 2020, 19, 1332; 32541938 10.1038/s41563-020-0710-z

[30] a) X. Tang , W. Liu , J. Wu , C.‐S. Lee , J. You , P. Wang , J. Org. Chem 2010, 75, 7273; 20939570 10.1021/jo101456v

[31] a) B. Kim , H. Shin , T. Park , H. Lim , E. Kim , Adv. Mater. 2013, 25, 5483; 23857668 10.1002/adma.201301834

[32] A. Leiter , R. R. Veluswamy , J. P. Wisnivesky , Nat. Rev. Clin. Oncol. 2023, 20, 624. 37479810 10.1038/s41571-023-00798-3

[33] a) F. Zeng , L. Tang , Q. Zhang , C. Shi , Z. Huang , S. Nijiati , X. Chen , Z. Zhou , Angew. Chem., Int. Ed. 2022, 61, e202112925; 10.1002/anie.202112925 34932846

[34] Y. Zheng , Y. Yan , L. Lin , Q. He , H. Hu , R. Luo , D. Xian , J. Wu , Y. Shi , F. Zeng , C. Wu , G. Quan , C. Lu , Acta Biomater. 2022, 142, 113. 35189382 10.1016/j.actbio.2022.02.019

