# Supplementary material for: Easy but Efficient: Facile Approach to Molecule with Theoretically Justified Donor–Acceptor Structure for Effective Photothermal Conversion and Intravenous Photothermal Therapy
Source: Adv Sci (Weinh). 2024 Mar 13;11(24):2309068. doi: 10.1002/advs.202309068 (PMC11200029; doi:10.1002/advs.202309068)
Supplement: Supplementary file 1 — Supporting Information [file ADVS-11-2309068-s001.pdf]

ADVANCED SCIENCE
Open Access

Supporting Information

for *Adv. Sci.*, DOI 10.1002/advs.202309068

Easy but Efficient: Facile Approach to Molecule with Theoretically Justified Donor–Acceptor Structure for Effective Photothermal Conversion and Intravenous Photothermal Therapy

Yuan-Hui Zhong, Gui-Feng Huang, Sheng-Yi Zhao, Lai-Hon Chung, Hua-Tang Zhang, Jin-Hong Zheng, Yi-Lang Yan, Wen-Xiu Ni and Jun He**

Supporting Information

Easy but Efficient: Facile Approach to Molecule with Theoretically Justified Donor-acceptor Structure for Effective Photothermal Conversion and Intravenous Photothermal Therapy

Yuan-Hui Zhong,[‡a] *Gui-Feng Huang,*[‡b] *Sheng-Yi Zhao,*[a] *Lai-Hon Chung,*[a] *Hua-Tang Zhang,*[a] *Jin-Hong Zheng,*[b] *Yi-Lang Yan,*[b] *Wen-Xiu Ni,**[b] *and Jun He**[a]

[a] School of Chemical Engineering and Light Industry, Guangdong University of Technology, Guangzhou 510006, P. R. China, Email: junhe@gdut.edu.cn

[b] Department of Medicinal Chemistry, Shantou University Medical College, Shantou, Guangdong, 515041, P. R. China, E-mail: wxni@stu.edu.cn

[‡] These authors contributed equally

Table of contents

General procedure **5**

Materials and characterization 5

Computational details 5

Experimental procedures **7**

Scheme S1. Synthetic scheme for D–A structured molecules. 7

Synthesis of *N*,*N*-dimethyl-4-((trimethylsilyl)ethynyl)aniline (**1-Me**) 7

Figure S1. The ^{1}H NMR spectrum of compound **1-Me** in $CDCl_3$ 8

Synthesis of *N*,*N*-diphenyl-4-((trimethylsilyl)ethynyl)aniline (**1-Ph**) 8

Figure S2. The ^{1}H NMR spectrum of compound **1-Ph** in $CDCl_3$ 8

Synthesis of 4-ethynyl-*N*,*N*-dimethylaniline (**2-Me**) 9

Figure S3. The ^{1}H NMR spectrum of compound **2-Me** in $CDCl_3$ 9

Synthesis of 4-ethynyl-*N*,*N*-diphenylaniline (**2-Ph**) 9

Figure S4. The ^{1}H NMR spectrum of compound **2-Ph** in $CDCl_3$ 10

Synthesis of 2-(4-(dimethylamino)phenyl)buta-1,3-diene-1,1,4,4-tetracarbonitrile (**DMA-TCNE**) 10

Figure S5. The ^{1}H NMR spectrum of compound **DMA-TCNE** in $CDCl_3$ 11

Figure S6. The MS spectrum of compound **DMA-TCNE** 11

Synthesis of 2-(4-(diphenylamino)phenyl)buta-1,3-diene-1,1,4,4-tetracarbonitrile (**DPA-TCNE**) 11

Figure S7. The ^{1}H NMR spectrum of compound **DPA-TCNE** in $CDCl_3$ 12

Figure S8. The MS spectrum of compound **DPA-TCNE** 12

Synthesis of 2-(4-(3,3-dicyano-1-(4-(dimethylamino)phenyl)allylidene)cyclohexa-2,5-dien-1-ylidene)malononitrile (**DMA-TCNQ**) 12

Figure S9. The ^{1}H NMR spectrum of compound **DMA-TCNQ** in $CDCl_3$ 13

Figure S10. The MS spectrum of compound **DMA-TCNQ** 13

Synthesis of 2-(4-(3,3-dicyano-1-(4-(diphenylamino)phenyl)allylidene)cyclohexa-2,5-dien-1-ylidene)malononitrile (**DPA-TCNQ**) 13

Figure S11. The ^{1}H NMR spectrum of compound **DPA-TCNQ** in $CDCl_3$ 14

Figure S12. The MS spectrum of compound **DPA-TCNQ** 14

Figure S13. Selected terminal electron donors (sky blue) and acceptors (orange) utilized in the *CA–RE* reaction. 15

Figure S14. The dipole moment of **DMA-TCNE**, **DPA-TCNE**, **DMA-TCNQ**, and **DPA-TCNQ**...... 15

Figure S15. The centroids of charge (C_{ele} and C_{hole}) of **DMA-TCNE**, **DPA-TCNE**, **DMA-TCNQ**, and **DPA-TCNQ** (the orange part depicts hole while green part refers to electron with isovalue of 0.002 au.). 16

Figure S16. TEM images of **DMA-TCNE** (a), **DPA-TCNE** (b), **DMA-TCNQ** (c), and **DPA-TCNQ** (d). (scale bar = 200 nm). 16

Figure S17. Temperature elevation plots of **DMA-TCNE** (a), **DPA-TCNE** (b), **DMA-TCNQ** (c), and **DPA-TCNQ** (d) in 1% DMSO/PBS solutions at different concentrations (5, 10, 20, 40, 80 μM) under NIR lamp irradiation (760 nm, 1.2 W cm^{-2}). 17

Figure S18. Temperature elevation plots of **DMA-TCNE** (a), **DPA-TCNE** (b), **DMA-TCNQ** (c), and **DPA-TCNQ** (d) in 1% DMSO/PBS solutions at a concentration of 80 μM under NIR lamp irradiation (760 nm) at varying power densities (0.2, 0.4, 0.6, 0.8, 1.0, 1.2 W cm^{-2}). 18

Figure S19. IR images of **DMA-TCNE**, **DPA-TCNE**, **DMA-TCNQ**, and **DPA-TCNQ** before and after irradiation with 760-nm NIR lamp (80 μM, 1.2 W cm^{-2}, 10 minutes). 18

Figure S20. The UV-vis-NIR spectra of D–A structured molecules (80 μM) before and after illumination with 1.2 W cm^{-1} for 10 minutes. 19

Figure S21. Hemolysis assays of **DMA-TCNQ**. (a) OD value of different groups (Inset: A photograph was taken after 24-hour incubation with **DMA-TCNQ**) (b) Hemolysis rate of various concentrations was calculated. 19

Figure S22. Infrared photographs of mice in the light group before and after individual treatments at day 0. 20

Figure S23. Infrared photographs of mice in the light group before and after individual treatments at day 8. 20

Figure S24. Photos of different groups of mice at day 14 after intravenous injection. (n = 6). 21

Figure S25. H&E-stained sections of the tumor tissues and major organs (heart, liver, spleen, kidney,

and lung) from mice after various treatments. Scale bar = 50 μm. 21

Supporting tables 22

Table S1. The optimized atomic coordinates and HOMO-LUMO orbital composition of **DMA-TCNE** in ground state.[a] 22

Table S2. The optimized atomic coordinates and HOMO-LUMO orbital composition of **DPA-TCNE** in ground state.[a] 23

Table S3. The optimized atomic coordinates and HOMO-LUMO orbital composition of **DMA-TCNQ** in ground state. [a] 24

Table S4. The optimized atomic coordinates and HOMO-LUMO orbital composition of **DPA-TCNQ** in ground state. [a] 25

Table S5. The fraction of electron, hole, overlap, and difference of D–A structured molecules in excited state........ 27

Table S6. The hole-electron index of D–A structured molecules in excited state. 27

Table S7. The calculation table of PCE with detailed parameters........ 28

Supporting references 29

General procedure

Materials and characterization

The starting materials, reagents, and solvents were procured from commercial sources (J&K, Zhengzhou Alfa, and Acros) and were used as received without any further purification. Solution ^{1}H NMR spectra were acquired using a 400 MHz Bruker superconducting magnet high-field Nuclear Magnetic Resonance (NMR) spectrometer at 298.15 K, with tetramethylsilane (TMS) serving as the internal standard. UV-Vis-NIR spectra were recorded using a UV-Visible Near Infra-Red Spectrophotometer with Integrating Sphere (Shimazu 3600 plus). The temperature change was monitored in real-time using an IR thermal camera (Thermo X, Shanghai Magnity Technologies Electronics Co. Ltd.). Dynamic light scattering (DLS) particle size analysis were performed using a Nanosight NS300HSBF instrument. The small animal phototherapy irradiator used was PR-LED5-760nm (Shenzhen Puri Materials Technologies, Co., Ltd.). For experimental animal studies, individually ventilated cages were utilized (Techniplast/IVC SealsafeTM, Italy). The vernier caliper employed was Digital Calipers 111-101-40 (Guilin Guanglu Measuring Instrument Co., Ltd.). The fluorescence spectra were measured using HORIBA Scientific Fluorolog-3 at room temperature. The mass spectra (ESI-MS) were collected by TSQ Endura Triple Quadrupole Mass Spectrometer (Thermo Fisher). The transmission electron microscope (TEM) images were collected by Hitachi HT7700.

Computational details

Electronic structure calculations in ground state of D–A structured molecules were performed using density functional theory (DFT) *via* Gaussian 09 software.[S1] The PBE0-D3 functional coupled with a 6-311g(d) basis set was utilized for optimizing the geometric structures. To ensure structural conformation accuracy, molecular

coordinates for structure optimization were obtained from previously reported crystallographic data.[S2-S4] Vibrational frequencies at the optimized structures were also calculated using the same DFT method to verify that the optimized structure represented the local minimal on the S_0 energy surface. The excited state electronic properties of D–A structured molecules were performed using TD-DFT with PBE0-D3 functional coupled with a 6-311g(d) basis set was utilized. The root mean square deviation (RMSD) between S_1 and S_0 structures was calculated by VMD 1.9.3.[S5] The radiative decay rate ($k_p = E^2 f/1.499\ s^{-1}$) was evaluated employing the Einstein spontaneous emission relationship, where E represents the vertical excitation energy, f denotes the oscillator strength. In addition, the reorganization energy and the nonradiative transition rates were calculated using the MOMAP package.[S6]

Experimental procedures

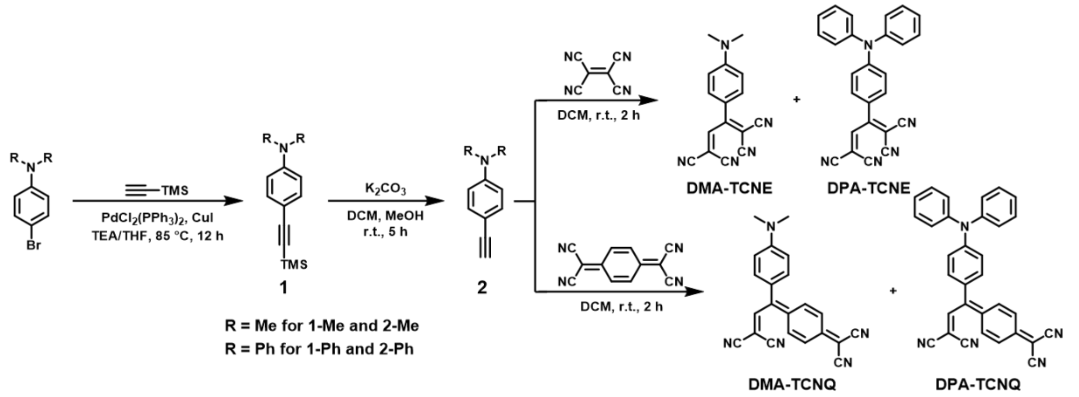

Scheme S1. Synthetic scheme for D–A structured molecules.

Synthesis of *N*,*N*-dimethyl-4-((trimethylsilyl)ethynyl)aniline (**1-Me**)

The synthesis of **1-Me** was achieved using a modified procedure of a previously reported protocol.[S7] In a 100 mL Schlenk tube, a mixture of 4-bromo-*N*,*N*-dimethylaniline (2.0 g, 10 mmol), bis(triphenylphosphine)palladium(II) chloride (351 mg, 0.5 mmol), and copper(I) iodide (190 mg, 1.0 mmol) were added and connected to a nitrogen manifold. Next, triethylamine (20.0 mL) and THF (20.0 mL), each previously purged by bubbling nitrogen gas for five minutes, were transferred into the tube. After injecting ethynyltrimethylsilane (4.1 mL, 30 mmol), the tube was screw-capped, and the reaction mixture was stirred and heated to reflux under nitrogen protection for 12 hours. The resulting mixture was cooled to room temperature, and the solvents were removed by rotary evaporation. The resulting residue was purified by column chromatography (eluent: hexanes/DCM, v/v = 12:1), resulting in **1-Me** as a yellow solid (1.95 g, 90.1% yield based on 4-bromo-*N*,*N*-dimethylaniline). ^{1}H NMR (400 MHz, Chloroform-*d*) δ 7.39 (d, J = 8.9 Hz, 2H), 6.61 (d, J = 8.9 Hz, 2H), 2.97 (s, 6H), 0.31 (s, 9H).

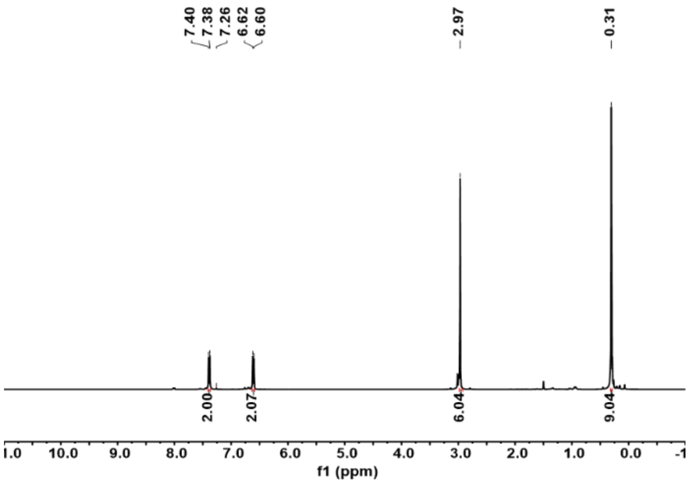

Figure S1. The ^{1}H NMR spectrum of compound **1-Me** in $CDCl_3$

Synthesis of *N*,*N*-diphenyl-4-((trimethylsilyl)ethynyl)aniline (**1-Ph**)

The synthesis of **1-Ph** followed a similar procedure to that of **1-Me**, with the exception of replacing the starting material 4-bromo-dimethylaniline with 4-bromo-*N*,*N*-diphenylaniline. After purification by column chromatography, a yellow solid **1-Ph** was obtained with a yield of 2.98 g (87.5% yield based on 4-bromo-*N*,*N*-diphenylaniline). ^{1}H NMR (400 MHz, Chloroform-*d*) δ 7.20 (d, J = 8.8 Hz, 2H), 7.11 (d, J = 8.0 Hz, 4H), 6.96 (t, 4H), 6.91 (d, J = 7.4 Hz, 2H), 6.84 (d, J = 8.9 Hz, 2H), 0.15 (s, 9H).

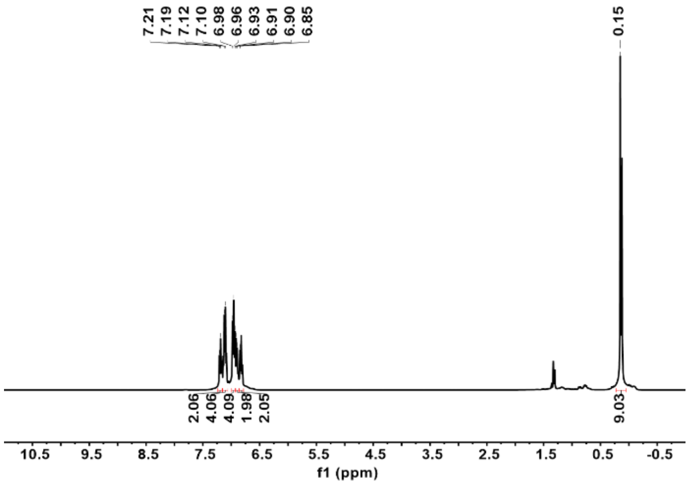

Figure S2. The ^{1}H NMR spectrum of compound **1-Ph** in $CDCl_3$

Synthesis of 4-ethynyl-*N*,*N*-dimethylaniline (**2-Me**)

2-Me was synthesized using a modified method of previously reported protocol.[S7] A mixture of **1-Me** (1.95 g, 8.9 mmol) and anhydrous potassium carbonate (2.48 g, 17.9 mmol) in a solution of DCM (20.0 mL) and methanol (20.0 mL) was stirred at room temperature for one hour. The reaction mixture was then filtered to remove the undissolved K_2CO_3, and the resulting organic filtrate was extracted with DCM (3 × 80 mL), washed with distilled water (3 × 100 mL), and dried over anhydrous $MgSO_4$. After solvent removal, the resulting orange solid was used directly in the next step (1.23 g, 95.6% yield based on **1-Me**). ^{1}H NMR (400 MHz, Chloroform-*d*) δ 7.40 (d, *J* = 8.6 Hz, 2H), 6.64 (d, *J* = 8.6 Hz, 2H), 3.01 (s, 1H), 2.98 (s, 6H).

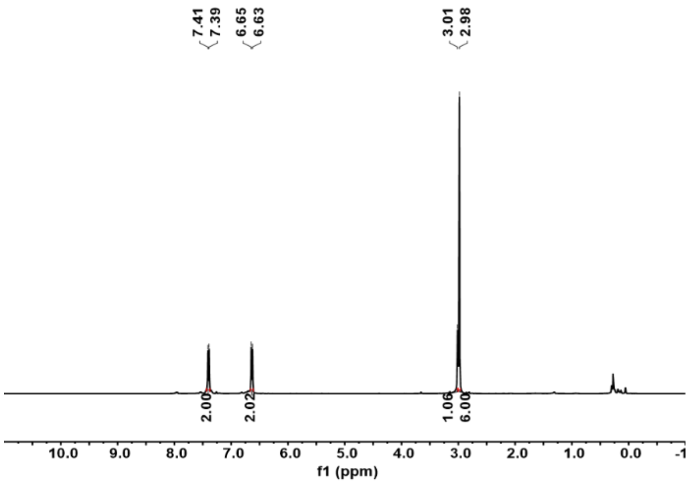

Figure S3. The ^{1}H NMR spectrum of compound **2-Me** in $CDCl_3$

Synthesis of 4-ethynyl-*N*,*N*-diphenylaniline (**2-Ph**)

The synthesis of **2-Ph** was carried out using a similar procedure to that of **2-Me**, with the exception of replacing the starting material **1-Me** with **1-Ph**. After the reaction, a yellow solid was obtained with a yield of 2.19 g (91.3% yield based on **1-Ph**). ^{1}H NMR (400 MHz, Chloroform-*d*) δ 7.22 (d, *J* = 8.1 Hz, 2H), 7.16 (t, *J* = 7.5 Hz, 4H), 6.99 (d, *J* = 8.2 Hz, 4H), 6.94 (d, *J* = 7.1 Hz, 2H), 6.86 (d, *J* = 8.0 Hz, 2H), 2.90 (s, 1H).

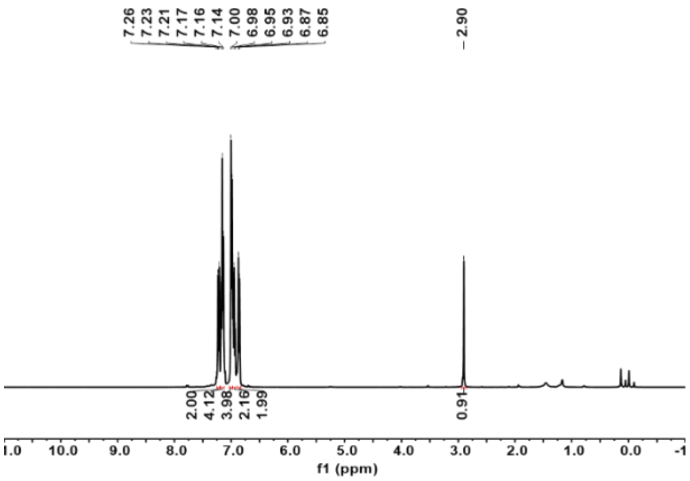

Figure S4. The ^{1}H NMR spectrum of compound **2-Ph** in $CDCl_3$

Synthesis of 2-(4-(dimethylamino)phenyl)buta-1,3-diene-1,1,4,4-tetracarbonitrile (**DMA-TCNE**)

DMA-TCNE was synthesized using a modified of method previously reported protocol.[S2] A solution of **2-Me** (435.0 mg, 3.0 mmol) in DCM (10 mL) was added to ethene-1,1,2,2-tetracarbonitrile (512.0 mg, 4.0 mmol), and the mixture was stirred for 2 hours at room temperature under a nitrogen atmosphere. The resulting reaction solution was concentrated under reduced pressure, followed by column chromatography packed with silica gel using petroleum ether/ethyl acetate (1:1, v/v) as the eluent. A pink solid, **DMA-TCNE**, was obtained in a yield of 714 mg (87.3% based on **2-Me**). ^{1}H NMR (400 MHz, Chloroform-*d*) δ 8.02 (s, 1H), 7.50 (d, J = 9.2 Hz, 2H), 6.76 (d, J = 9.2 Hz, 2H), 3.17 (s, 7H). MS (ESI-MS): found = 274.02, calculated = 274.11 (formula: M + H^+)

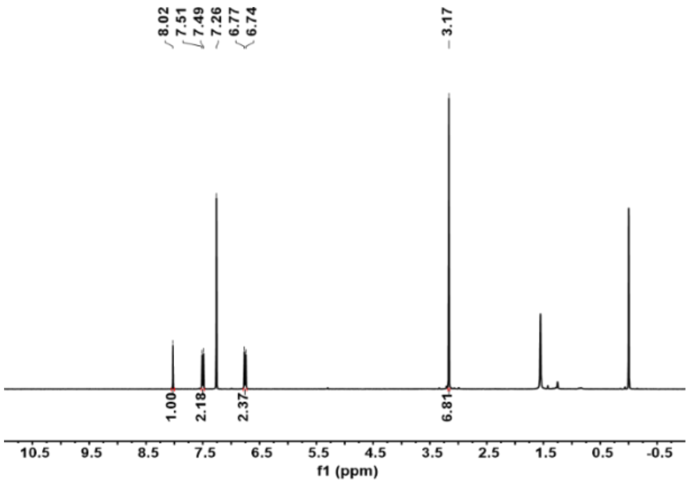

Figure S5. The ^{1}H NMR spectrum of compound **DMA-TCNE** in $CDCl_3$

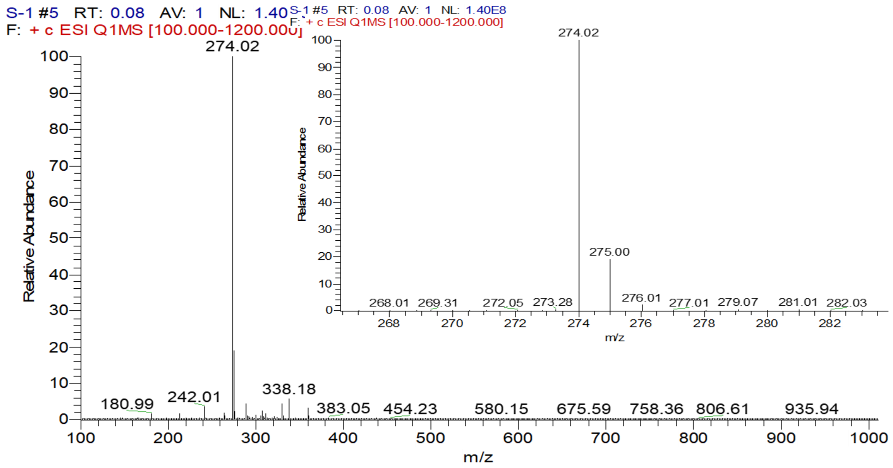

Figure S6. The MS spectrum of compound **DMA-TCNE**

Synthesis of 2-(4-(diphenylamino)phenyl)buta-1,3-diene-1,1,4,4-tetracarbonitrile (**DPA-TCNE**)

The synthesis of **DPA-TCNE** followed a similar procedure to that of **DMA-TCNE**, with the exception of **2-Ph** replaced by **2-Me** as the starting material. After purification by column chromatography, a purple solid was yielded (969.5 mg, 81.4% based on **2-Ph**). ^{1}H NMR (400 MHz, Chloroform-*d*) δ 8.01 (s, 1H), 7.39 (t, J = 7.6 Hz, 4H), 7.34 (d, J = 7.1 Hz, 2H), 7.24 (d, J = 7.8 Hz, 4H), 7.21 (d, J = 7.8 Hz, 3H), 7.03 (d, J = 7.2 Hz, 2H). MS (ESI-MS): found = 398.08, calculated = 398.14 (formula: $M + H^+$)

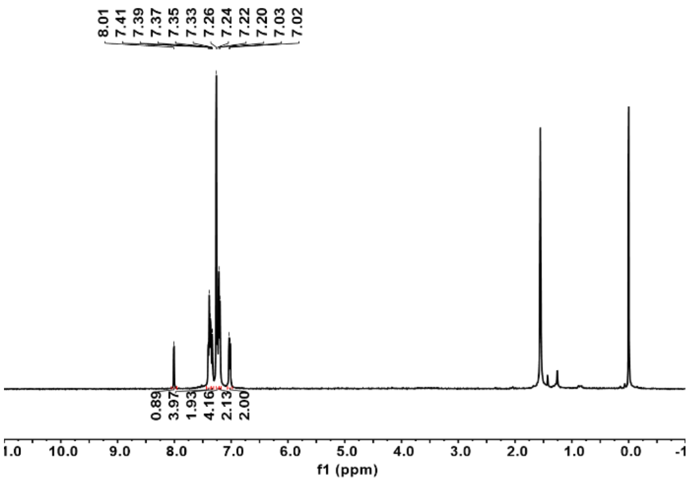

Figure S7. The ^{1}H NMR spectrum of compound **DPA-TCNE** in $CDCl_3$

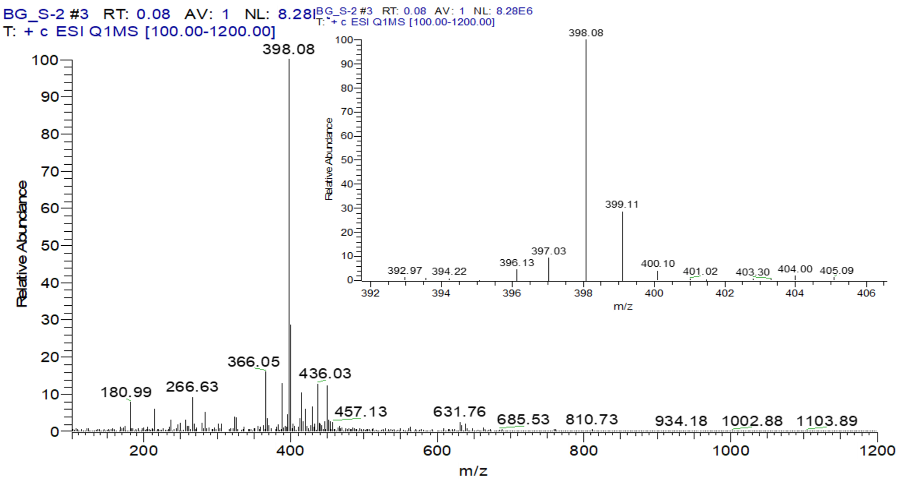

Figure S8. The MS spectrum of compound **DPA-TCNE**

Synthesis of 2-(4-(3,3-dicyano-1-(4-(dimethylamino)phenyl)allylidene)cyclohexa-2,5-dien-1-ylidene)malononitrile (**DMA-TCNQ**)

The synthesis of **DMA-TCNQ** was analogous to that of **DMA-TCNE**, with the replacement of the starting material, ethene-1,1,2,2-tetracarbonitrile, by 7,7,8,8-Tetracyanoquinodimethane. Following purification *via* column chromatography, a fuchsia solid was isolated (819.8 mg, 78.3% yield based on **2-Me**). ^{1}H NMR (400 MHz, Chloroform-*d*) δ 8.18 (s, 1H), 7.29 (d, J = 20.7 Hz, 4H), 7.16 (d, J = 8.9 Hz, 2H), 6.82 (d, J = 8.5 Hz, 2H), 3.15 (s, 6H). MS (ESI-MS): found = 350.02, calculated = 350.14 (formula: M + H^+)

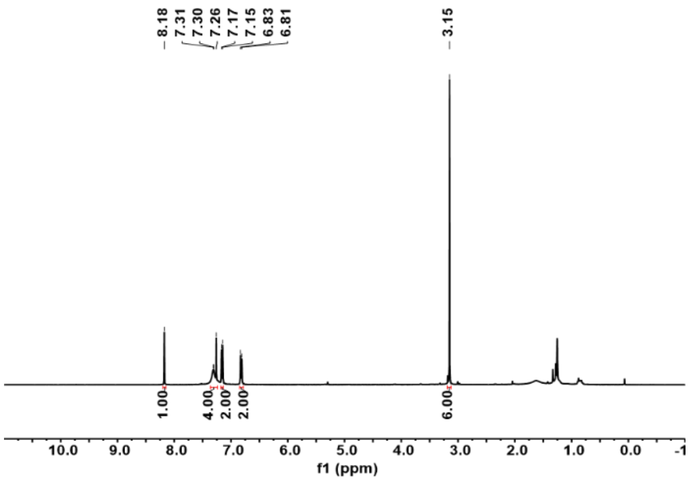

Figure S9. The ^{1}H NMR spectrum of compound **DMA-TCNQ** in $CDCl_3$

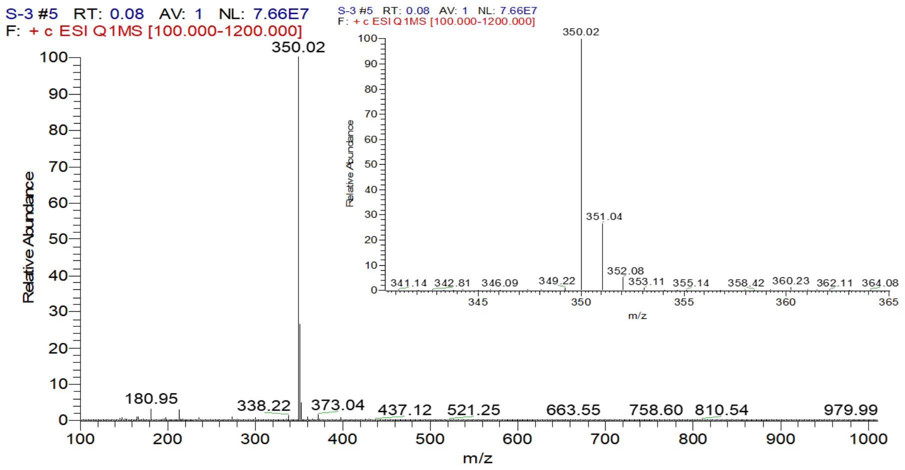

Figure S10. The MS spectrum of compound **DMA-TCNQ**

Synthesis of 2-(4-(3,3-dicyano-1-(4-(diphenylamino)phenyl)allylidene)cyclohexa-2,5-dien-1-ylidene)malononitrile (**DPA-TCNQ**)

The synthesis of **DPA-TCNQ** followed a procedure analogous to that of **DMA-TCNQ**, with the substitution of the starting material, **2-Me**, by **2-Ph**. Following purification *via* column chromatography, a black solid was obtained (1038.9 mg, 73.2% yield based on **2-Ph**). ^{1}H NMR (400 MHz, Chloroform-*d*) δ 8.18 (s, 1H), 7.56 (s, 2H), 7.38 (d, J = 10.7 Hz, 2H), 7.33 (t, J = 15.7 Hz, 4H), 7.21 (d, J = 8.0 Hz, 4H), 7.16 (d, J = 7.2 Hz, 2H), 7.12(d, J = 7.2 Hz, 2H), 7.05 (d, J = 8.6 Hz, 2H). MS (ESI-MS): found = 474.09, calculated = 474.17 (formula: M + H^+)

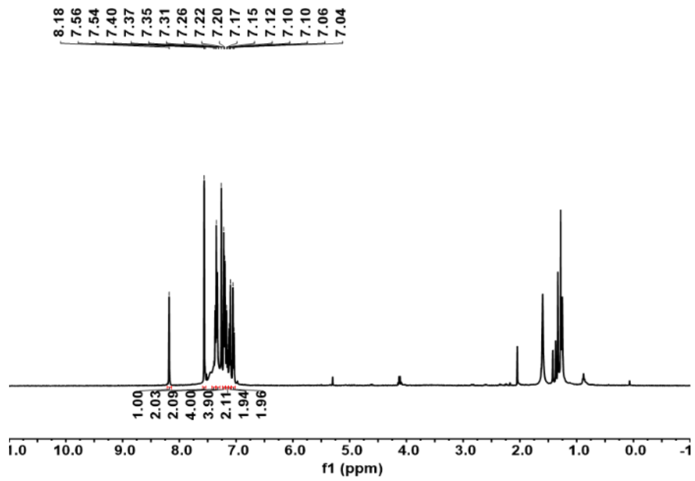

Figure S11. The ^{1}H NMR spectrum of compound **DPA-TCNQ** in $CDCl_3$

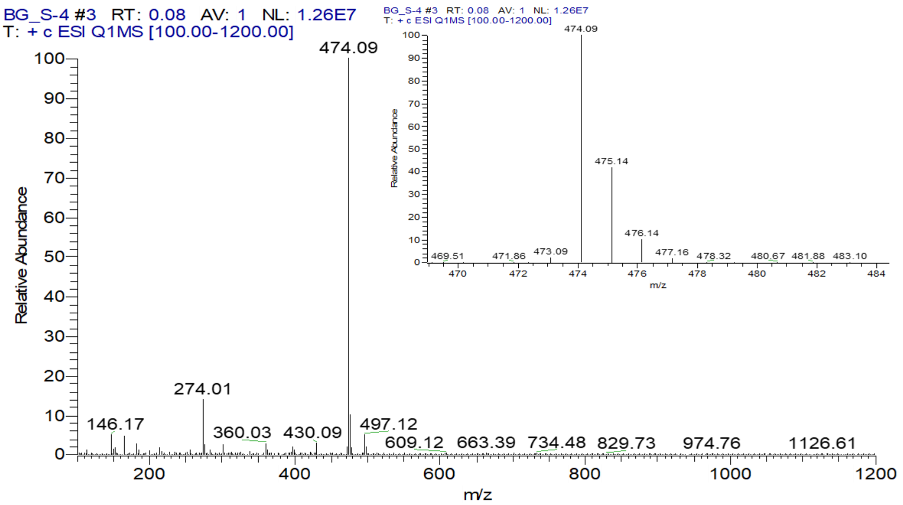

Figure S12. The MS spectrum of compound **DPA-TCNQ**

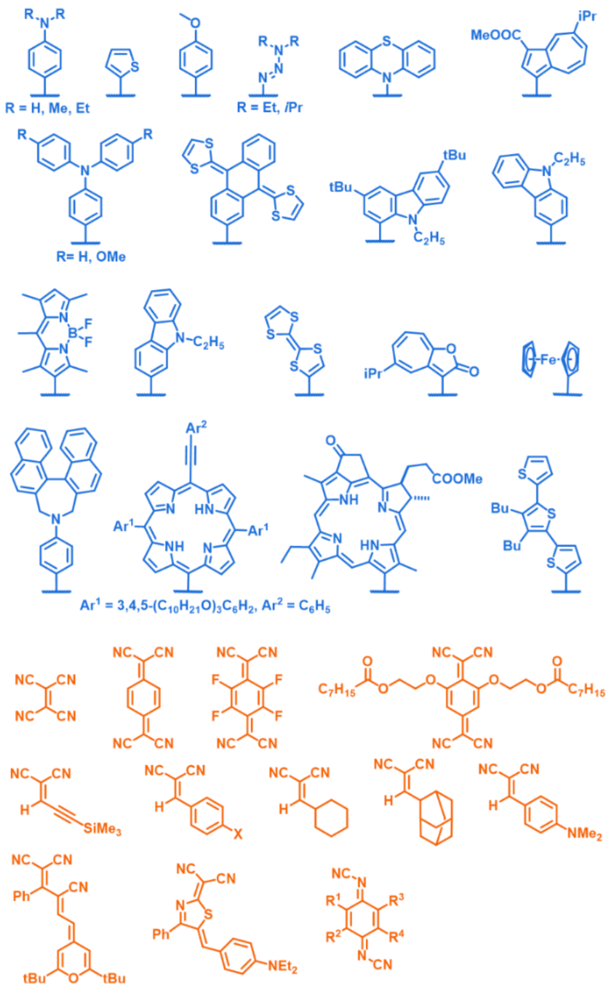

Figure S13. Selected terminal electron donors (sky blue) and acceptors (orange) utilized in the *CA–RE* reaction.

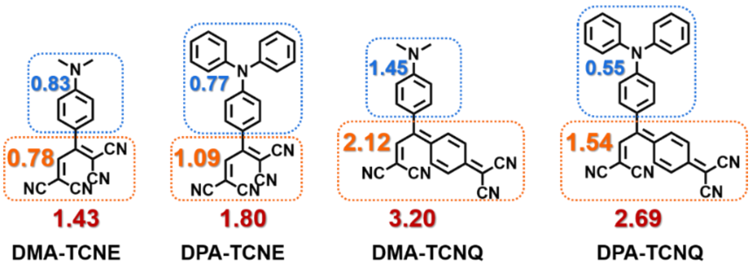

Figure S14. The dipole moment of **DMA-TCNE**, **DPA-TCNE**, **DMA-TCNQ**, and **DPA-TCNQ**.

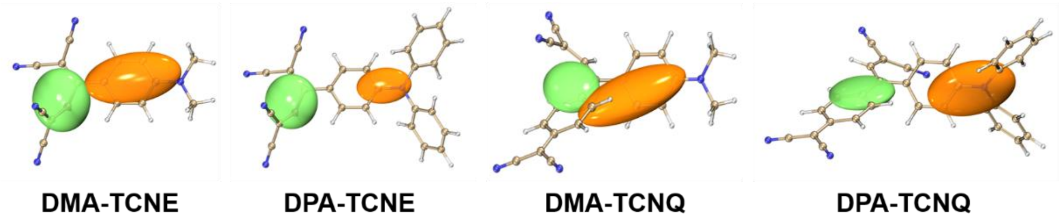

Figure S15. The centroids of charge (C_{ele} and C_{hole}) of **DMA-TCNE**, **DPA-TCNE**, **DMA-TCNQ**, and **DPA-TCNQ** (the orange part depicts hole while green part refers to electron with isovalue of 0.002 au.).

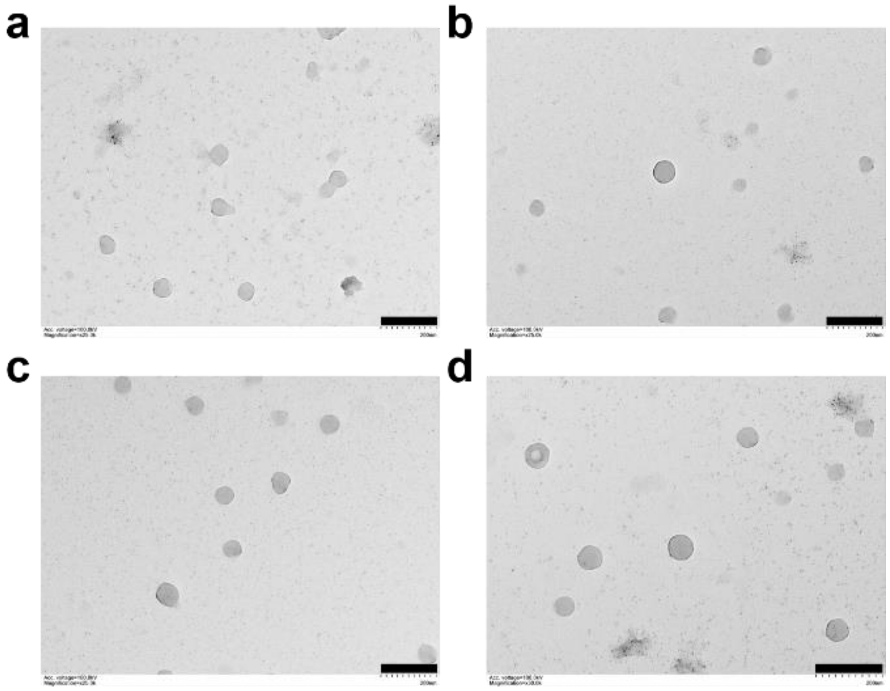

Figure S16. TEM images of **DMA-TCNE** (a), **DPA-TCNE** (b), **DMA-TCNQ** (c), and **DPA-TCNQ** (d) (scale bar = 200 nm).

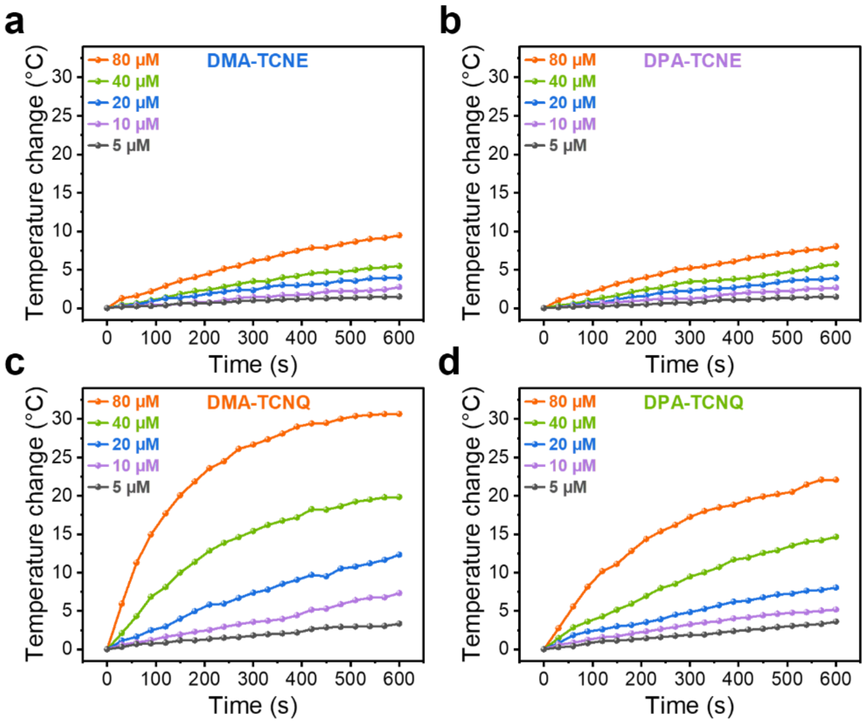

Figure S17. Temperature elevation plots of **DMA-TCNE** (a), **DPA-TCNE** (b), **DMA-TCNQ** (c), and **DPA-TCNQ** (d) in 1% DMSO/PBS solutions at different concentrations (5, 10, 20, 40, 80 μM) under NIR lamp irradiation (760 nm, 1.2 W cm^{-2}).

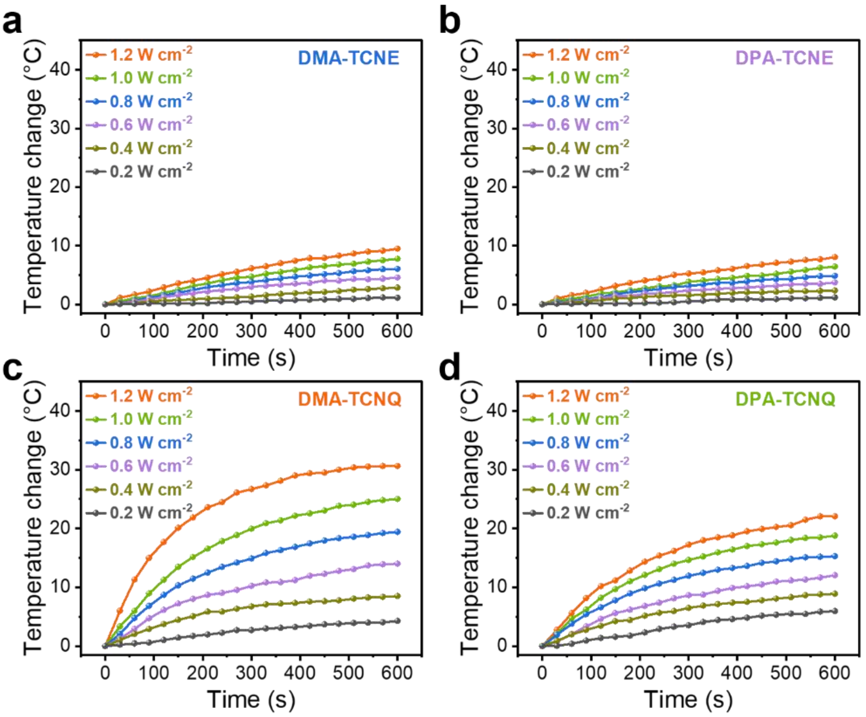

Figure S18. Temperature elevation plots of **DMA-TCNE** (a), **DPA-TCNE** (b), **DMA-TCNQ** (c), and **DPA-TCNQ** (d) in 1% DMSO/PBS solutions at a concentration of 80 μM under 760-nm LED lamp irradiation at varying power densities (0.2, 0.4, 0.6, 0.8, 1.0, 1.2 W cm^{-2}).

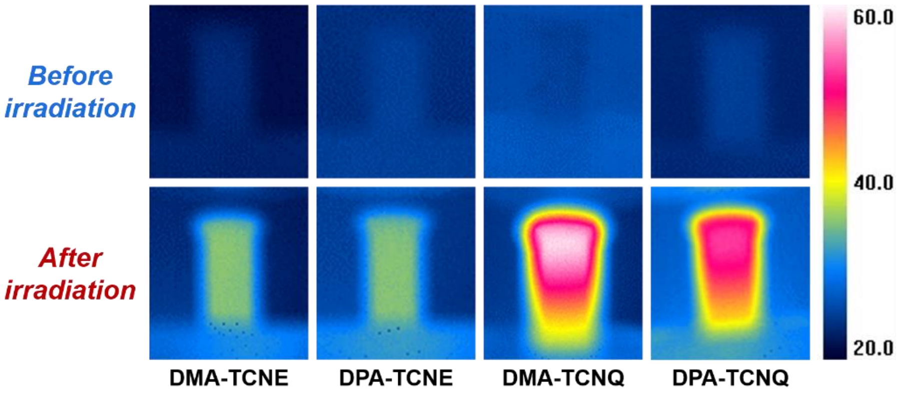

Figure S19. IR images of **DMA-TCNE**, **DPA-TCNE**, **DMA-TCNQ**, and **DPA-TCNQ** before and after irradiation with a 760-nm LED lamp (80 μM in 1% DMSO PBS solution, 1.2 W cm^{-2}, 10 minutes).

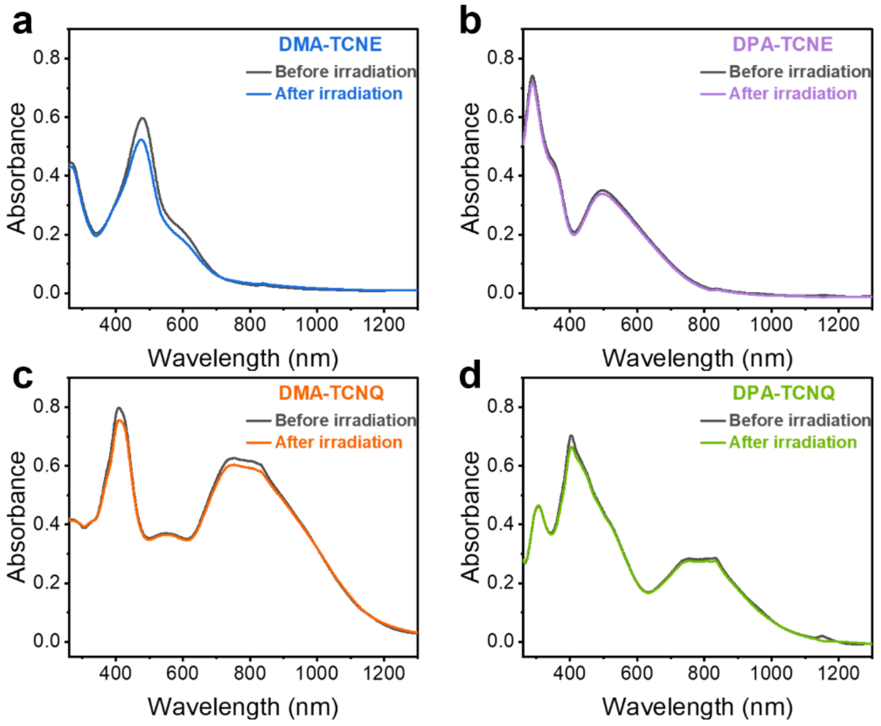

Figure S20. The UV-vis-NIR spectra of D–A structured molecules (80 μM) before and after illumination with 1.2 W cm^{-1} for 10 minutes.

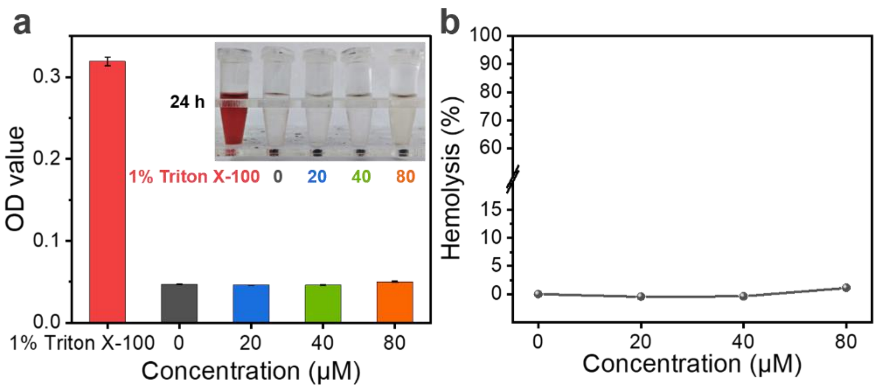

Figure S21. Hemolysis assays of **DMA-TCNQ**. (a) OD value of different groups (Inset: A photograph was taken after 24-hour incubation with **DMA-TCNQ**) (b) Hemolysis rate of various concentrations was calculated.

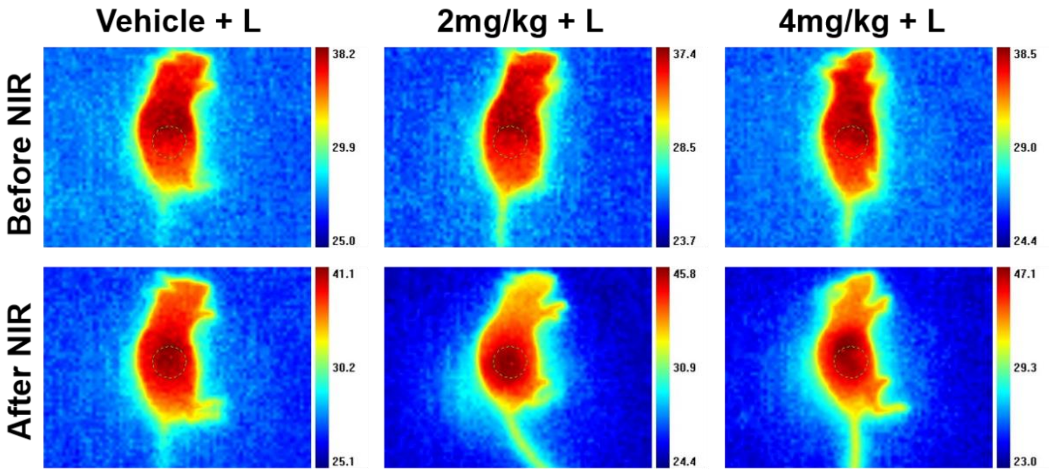

Figure S22. Infrared photographs of mice in the light group before and after individual treatments at day 0.

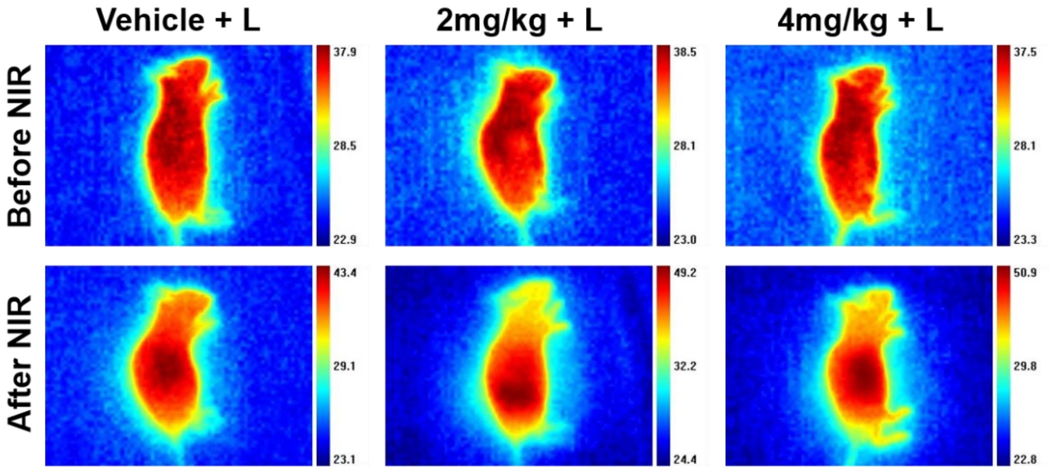

Figure S23. Infrared photographs of mice in the light group before and after individual treatments at day 8.

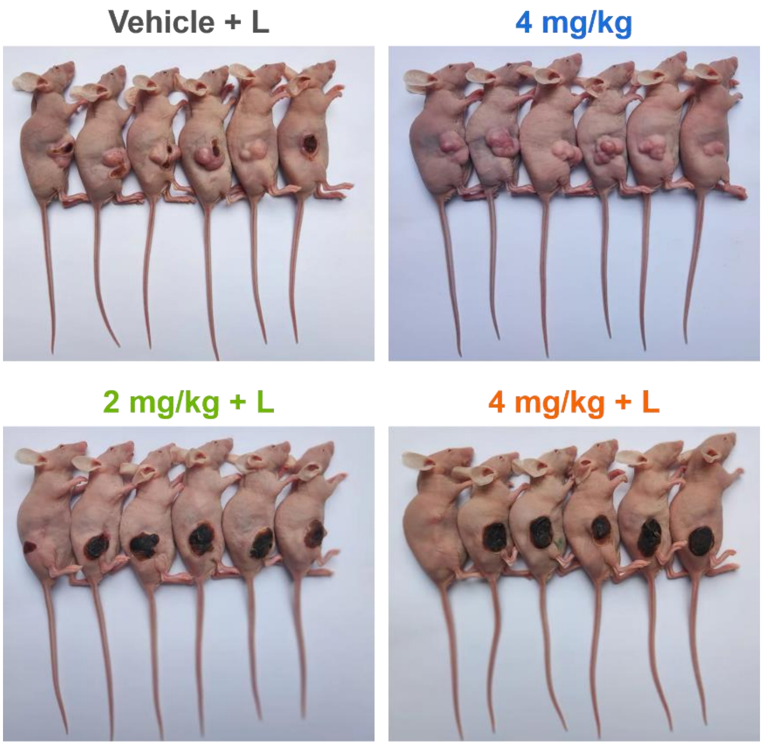

Figure S24. Photos of different groups of mice at day 14 after intravenous injection. (n = 6).

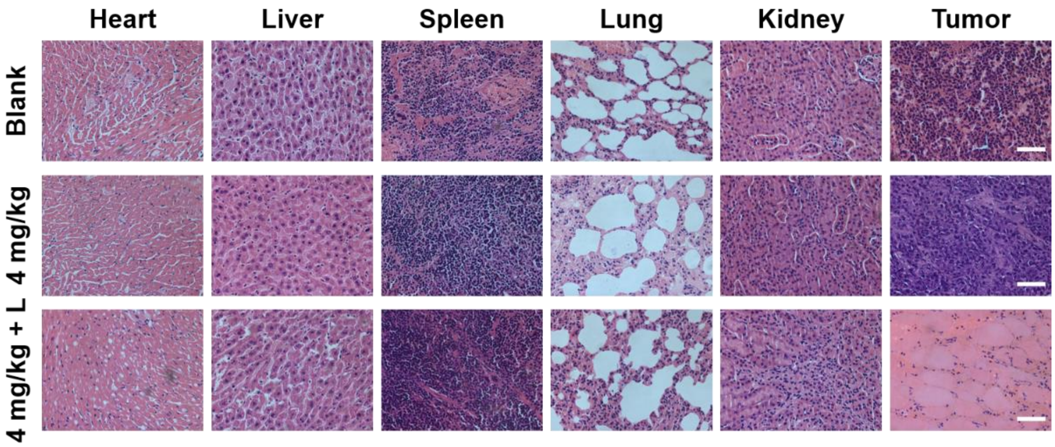

Figure S25. H&E-stained sections of the tumor tissues and major organs (heart, liver, spleen, kidney, and lung) from mice after various treatments (scale bar = 50 μm).

Supporting tables

Table S1. The optimized atomic coordinates and HOMO-LUMO orbital composition of **DMA-TCNE** in ground state.[a]

Atom	Coordinate			Orbital composition (%)	
	x	y	z	HOMO	LUMO
C1	2.678328	0.987121	-0.42504	8.451	0.464
C2	1.3275	1.218698	-0.53018	2.921	1.62
C3	0.383732	0.209015	-0.26849	12.671	2.28
C4	0.885274	-1.05636	0.086838	3.08	2.195
C5	2.233518	-1.29935	0.210555	9.135	0.737
C6	3.179753	-0.27764	-0.03578	7.006	1.511
N7	4.514047	-0.50238	0.08779	22.651	1.193
C8	-1.04917	0.404991	-0.37025	2.415	12.585
C9	5.456985	0.567125	-0.15303	2.873	0.156
C10	4.997912	-1.8101	0.468979	2.853	0.156
C11	-1.71765	1.593105	-0.20211	6.975	10.8
C12	-1.82198	-0.79171	-0.73967	0.464	16.085
C13	-2.81481	-1.34584	-0.01544	0.16	12.793
C14	-3.10702	1.722681	-0.48838	1.453	2.996
N15	-4.22946	1.850452	-0.72782	3.471	3.967
C16	-1.09287	2.789763	0.248116	1.368	3.317
N17	-0.61917	3.775941	0.618401	2.945	5.174
C18	-3.46787	-2.52943	-0.47879	0.028	3.894
N19	-3.98055	-3.49154	-0.85464	0.05	5.719
C20	-3.23472	-0.8427	1.253829	0.1	3.721
N21	-3.56852	-0.46832	2.291912	0.131	5.365
H22	3.356493	1.798413	-0.65659	0.773	0.057
H23	1.002369	2.199975	-0.85279	0.162	0.19
H24	0.194914	-1.8654	0.307581	0.167	0.616
H25	2.555878	-2.2883	0.510096	0.843	0.056
H26	5.283235	1.416468	0.516726	1.56	0.079
H27	6.466933	0.203603	0.025843	0.166	0.014
H28	5.408378	0.926936	-1.18725	1.651	0.086
H29	4.698486	-2.57864	-0.25261	1.578	0.086
H30	6.085326	-1.79531	0.506238	0.163	0.011

H31	4.632514	-2.10497	1.459358	1.629	0.086
H32	-1.5205	-1.29803	-1.65303	0.108	1.991

[a]The composition analysis based on Hirshfeld method.

Table S2. The optimized atomic coordinates and HOMO-LUMO orbital composition of **DPA-TCNE** in ground state.[a]

**Atom**	**Coordinate**			**Orbital composition (%)**	
	**x**	**y**	**z**	**HOMO**	**LUMO**
C1	-0.65636	-1.18054	-0.70878	5.659	0.469
C2	0.7101	-1.31485	-0.7956	1.631	1.647
C3	1.57384	-0.30086	-0.34517	7.501	2.316
C4	0.986133	0.865356	0.176792	1.725	2.18
C5	-0.37992	1.002525	0.282643	5.988	0.643
C6	-1.2367	-0.02436	-0.15348	4.481	1.708
N7	-2.61039	0.099587	-0.04762	19.155	0.929
C8	-3.4479	-1.04577	0.022036	4.74	0.145
C9	-3.23012	1.376334	0.007872	4.468	0.131
C10	3.020996	-0.39933	-0.41931	1.481	12.85
C11	3.74507	-1.56361	-0.3558	3.794	11.308
C12	3.726859	0.872983	-0.62776	0.284	15.172
C13	4.682795	1.3892	0.171334	0.071	12.48
C14	-4.59025	-1.11683	-0.77417	3.885	0.154
C15	-5.42479	-2.22255	-0.69004	1.415	0.042
C16	-5.12265	-3.26735	0.176404	4.998	0.163
C17	-3.98262	-3.19509	0.969521	1.346	0.048
C18	-3.1512	-2.08578	0.903263	3.926	0.162
C19	-4.17983	1.643955	0.992774	3.718	0.125
C20	-4.80511	2.882373	1.033009	1.303	0.035
C21	-4.48261	3.863812	0.102141	4.649	0.125
C22	-3.53519	3.595402	-0.87988	1.257	0.041
C23	-2.91627	2.354354	-0.93599	3.734	0.132
C24	5.146019	-1.59709	-0.61166	0.785	3.099
N25	6.27877	-1.64592	-0.8302	1.796	4.256
C26	3.168078	-2.82818	-0.04803	0.769	3.398
N27	2.735938	-3.86765	0.209763	1.609	5.328
C28	5.267715	2.656122	-0.1362	0.012	3.728
N29	5.72609	3.684224	-0.38657	0.016	5.546

C30	5.124606	0.760438	1.375213	0.046	3.562
N31	5.473668	0.281742	2.364292	0.056	5.195
H32	-1.29519	-1.96848	-1.09011	0.462	0.056
H33	1.110931	-2.2063	-1.26238	0.107	0.194
H34	1.617656	1.663808	0.555279	0.107	0.585
H35	-0.79778	1.899066	0.725073	0.49	0.034
H36	3.399413	1.472826	-1.47293	0.069	1.89
H37	-4.81872	-0.30045	-1.45162	0.284	0.015
H38	-6.312	-2.271	-1.31349	0.131	0.003
H39	-5.77412	-4.13311	0.235622	0.439	0.017
H40	-3.7432	-4.0011	1.655816	0.12	0.006
H41	-2.26839	-2.0198	1.531081	0.29	0.025
H42	-4.42463	0.875185	1.718435	0.276	0.013
H43	-5.54285	3.083667	1.803289	0.124	0.003
H44	-4.97008	4.832556	0.139408	0.41	0.013
H45	-3.28574	4.351324	-1.61785	0.115	0.006
H46	-2.18806	2.133885	-1.71006	0.281	0.026

[a]The composition analysis based on Hirshfeld method.

Table S3. The optimized atomic coordinates and HOMO-LUMO orbital composition of **DMA-TCNQ** in ground state.[a]

Atom	Coordinate			Orbital composition (%)	
	x	y	z	HOMO	LUMO
C1	3.087963	-1.66156	-0.67869	4.082	0.447
C2	1.873708	-1.01566	-0.73079	2.492	1.512
C3	1.691103	0.270535	-0.19053	7.179	1.95
C4	2.818114	0.881028	0.387137	2.523	1.806
C5	4.03722	0.245272	0.458248	4.684	0.905
C6	4.211083	-1.05606	-0.0683	4.512	1.465
N7	5.411503	-1.69349	-0.00079	10.774	1.386
C8	5.558211	-3.02393	-0.54533	1.334	0.176
C9	6.553705	-1.03104	0.586478	1.329	0.176
C10	0.416749	0.966713	-0.26737	4.827	10.539
C11	-0.81176	0.301942	-0.18064	5.299	6.136
C12	-0.92337	-0.97966	0.459043	1.718	3.499
C13	-2.10415	-1.63917	0.53783	4.048	3.667
C14	-3.29367	-1.10499	-0.05959	2.277	5.13

C15	-3.18979	0.169524	-0.70902	3.912	3.29
C16	-2.01429	0.840486	-0.75046	1.628	3.169
C17	-4.49892	-1.79533	-0.0083	11.39	8.954
C18	0.504222	2.395481	-0.47947	1.168	8.753
C19	-0.27136	3.364374	0.082508	1.787	9.489
C20	-5.67983	-1.26768	-0.58577	2.293	2.126
N21	-6.63144	-0.82213	-1.06909	5.318	3.824
C22	-4.59562	-3.06302	0.616596	2.282	2.135
N23	-4.64821	-4.09802	1.130298	5.262	3.794
C24	-0.02997	4.733781	-0.23469	0.37	2.541
N25	0.176291	5.83893	-0.49675	0.789	4.223
C26	-1.29085	3.11606	1.046649	0.471	2.335
N27	-2.10794	2.947044	1.84426	0.754	3.458
H28	3.17328	-2.63824	-1.13809	0.375	0.057
H29	1.047022	-1.49753	-1.24308	0.169	0.172
H30	2.721936	1.868691	0.829788	0.156	0.364
H31	4.861836	0.75858	0.936408	0.434	0.076
H32	4.867625	-3.73088	-0.07238	0.703	0.088
H33	5.384451	-3.0442	-1.62789	0.787	0.102
H34	6.570941	-3.37833	-0.36354	0.08	0.014
H35	6.806694	-0.10756	0.052808	0.739	0.099
H36	6.381529	-0.78401	1.640332	0.749	0.096
H37	7.417677	-1.69088	0.53743	0.077	0.011
H38	-0.05138	-1.38736	0.957502	0.19	0.235
H39	-2.16727	-2.57595	1.081864	0.337	0.284
H40	-4.07036	0.588585	-1.18443	0.318	0.234
H41	-1.96727	1.783889	-1.28291	0.127	0.29
H42	1.330871	2.746292	-1.09272	0.258	0.995

[a]The composition analysis based on Hirshfeld method.

Table S4. The optimized atomic coordinates and HOMO-LUMO orbital composition of **DPA-TCNQ** in ground state.[a]

Atom	Coordinate			Orbital composition (%)	
	x	y	z	HOMO	LUMO
C1	-1.61597	0.366077	-1.29865	4.857	0.558
C2	-0.32721	0.843588	-1.39679	1.465	1.002
C3	0.605416	0.638235	-0.37052	6.171	1.731

C4	0.204356	-0.10142	0.750445	1.479	1.129
C5	-1.09356	-0.54907	0.874998	5.098	0.362
C6	-2.03515	-0.30291	-0.13736	4.221	1.081
N7	-3.35972	-0.68873	0.031604	18.561	0.985
C8	-4.17275	-1.01655	-1.07861	4.42	0.137
C9	-3.95271	-0.61703	1.319672	4.498	0.135
C10	1.932941	1.256498	-0.43396	1.033	10.188
C11	3.101714	0.507696	-0.2893	1.971	6.107
C12	1.996847	2.678058	-0.63148	0.346	8.007
C13	1.012811	3.58813	-0.3646	0.18	9.668
C14	-5.47032	-0.5115	-1.17562	4.033	0.145
C15	-6.26737	-0.85098	-2.25961	1.372	0.042
C16	-5.78008	-1.68291	-3.26166	5.102	0.16
C17	-4.48623	-2.1839	-3.16591	1.35	0.041
C18	-3.68763	-1.86312	-2.0777	3.984	0.16
C19	-4.75461	-1.66332	1.774298	3.722	0.119
C20	-5.3526	-1.58324	3.02418	1.348	0.034
C21	-5.14587	-0.46993	3.83173	4.78	0.127
C22	-4.34344	0.57091	3.37612	1.263	0.034
C23	-3.75303	0.508394	2.121426	3.872	0.134
C24	3.063641	-0.92874	-0.33777	0.459	3.695
C25	4.177404	-1.68198	-0.17605	1.112	3.76
C26	5.454803	-1.07422	0.063668	0.626	5.577
C27	5.506186	0.360143	0.105075	1.145	3.649
C28	4.390282	1.106885	-0.06816	0.381	3.694
C29	6.600076	-1.84091	0.232941	2.896	9.616
C30	1.234392	4.961638	-0.67427	0.034	2.457
N31	1.421218	6.071846	-0.93115	0.07	4.278
C32	-0.22652	3.30317	0.28238	0.21	2.296
N33	-1.22955	3.154341	0.835238	0.327	3.788
C34	7.865607	-1.24246	0.452389	0.586	2.285
N35	8.888001	-0.73247	0.631082	1.278	4.084
C36	6.553678	-3.25658	0.191148	0.585	2.284
N37	6.491906	-4.4107	0.152918	1.276	4.076
H38	-2.32601	0.551711	-2.09612	0.392	0.044
H39	-0.03516	1.40744	-2.27795	0.089	0.203
H40	0.911421	-0.27591	1.555569	0.107	0.113
H41	-1.40232	-1.07627	1.770509	0.399	0.045

H42	2.915133	3.098294	-1.03027	0.048	0.712
H43	-5.84618	0.144793	-0.39778	0.311	0.012
H44	-7.27459	-0.45152	-2.3256	0.122	0.003
H45	-6.40541	-1.94208	-4.10976	0.453	0.016
H46	-4.09881	-2.84372	-3.93603	0.11	0.005
H47	-2.6844	-2.26829	-1.99157	0.298	0.023
H48	-4.9067	-2.53129	1.140759	0.272	0.011
H49	-5.9756	-2.40122	3.372353	0.119	0.003
H50	-5.61069	-0.41248	4.810746	0.423	0.012
H51	-4.18515	1.448981	3.99435	0.118	0.005
H52	-3.13858	1.32747	1.758615	0.279	0.022
H53	2.116803	-1.41327	-0.54669	0.135	0.277
H54	4.117455	-2.76311	-0.24744	0.093	0.274
H55	6.459647	0.843869	0.290704	0.095	0.256
H56	4.475562	2.184773	0.012993	0.022	0.342

[a]The composition analysis based on Hirshfeld method.

Table S5. The fraction of electron, hole, overlap, and difference of D–A structured molecules in excited state.

Compounds	Fragment	Hole (%)	Electron (%)	Overlap (%)	Difference (%)
**DMA-TCNE**	D	80.34	7.89	25.17	-72.46
	A	19.66	92.11	42.55	72.46
**DPA-TCNE**	D	89.01	9.8	29.53	-79.21
	A	19.99	90.2	31.49	79.21
**DMA-TCNQ**	D	65.46	9.19	24.53	-56.26
	A	34.54	90.81	56.01	56.26
**DPA-TCNQ**	D	91.73	8.14	27.33	-83.59
	A	8.27	91.86	27.56	83.59

Table S6. The hole-electron index of D–A structured molecules in excited state.

Compounds	*D* (Å)	S_r	*H* (Å)	*t*-index (Å)
**DMA-TCNE**	4.08	0.42	2.78	2.03
**DPA-TCNE**	5.42	0.39	3.05	3.16
**DMA-TCNQ**	3.28	0.62	3.86	0.48
**DPA-TCNQ**	5.88	0.40	3.70	3.10

Table S7. The calculation table of PCE with detailed parameters.

Compounds	DMA-TCNE	DPA-TCNE	DMA-TCNQ	DPA-TCNQ
m (g)	1.5	1.5	1.5	1.5
C_p (J g^{-1} °C^{-1})	4.18	4.18	4.18	4.18
T_{max} (°C)	36.80	36.28	59.09	50.57
T_{surr} (°C)	27.35	28.26	28.44	28.49
ΔT (°C)	9.45	8.02	30.65	22.08
I (W cm^{-2})	1.2	1.2	1.2	1.2
A_{760}	0.05232	0.06538	0.54295	0.31463
τ_s	493.3	380.3	245.9	302.8
η (%)	88.2	78.9	91.3	79.9

Table S8. The photothermal conversion efficiency (PCE) of small-molecular organic PTAs in the reported literatures.

Compounds	Laser (nm)	Power (W cm^{-2})	PCE (%)	Reference
**DMA-TCNQ**	**760**	**1.2**	**91.28**	**This work**
tmf-BDP	808	0.3	88.30	*Adv. Mater.* **2020**, *32*, 1907855
**DMA-TCNE**	**760**	**1.2**	**88.20**	**This work**
CF3cy	808	0.5	83.00	*Adv. Funct. Mater.* **2023**, 2300340
FE-IDMN	808	1.0	82.60	*Adv. Funct. Mater.* **2023**, 2311365
ICR-Qu	808	1.0	81.10	*Adv. Mater.* **2023**, 2210179
BAF4	1064	0.8	80.00	*Angew. Chem. Int. Ed.* **2021**, *60*,22376
**DPA-TCNQ**	**760**	**1.2**	**79.93**	**This work**
**DPA-TCNE**	**760**	**1.2**	**78.88**	**This work**
FE-BA	808	1.0	73.90	*Adv. Funct. Mater.* **2023**, 2311365
TPTQ	808	0.5	73.32	*Chem. Eng. J.* **2023** 468, 143726
CNcy	808	0.5	62.40	*Adv. Funct. Mater.* **2023**, 2300340
BD3	915	0.6	60.30	*Angew. Chem. Int. Ed.* **2022**, *61*, e202211081
B4-BSA	808	1.5	60.02	*Adv. Mater.* **2022**, 34, 2109111
Ecy	808	0.5	54.00	*Adv. Funct. Mater.* **2023**, 2300340
NM	808	1.0	47.40	*Biomaterials* **2022**, 287, 121670
DTTVBI	808	0.8	45.80	*J. Am. Chem. Soc.* **2023**, 145, 334
IR-FE-TPP	808	1.0	43.90	*Small* **2023**, 2207995
DPBTA-DPTQ	808	0.2	40.60	*Angew. Chem. Int. Ed.* **2021**, 60, 26769
BT6	808	1.0	36.00	*Adv. Mater.* **2023**, 2211632
TPA-BT-DPTQ	808	0.8	23.00	*Angew. Chem. Int. Ed.* **2022**, 61, e202202614

Supporting references

[S1] M. J. Frisch, G. W. Trucks, H. B. Schlegel, G. E. Scuseria, M. A. Robb, J. R. Cheeseman, G. Scalmani, V. Barone, G. A. Petersson, H. Nakatsuji, X. Li, M. Caricato, A. Marenich, J. Bloino, B. G. Janesko, R. Gomperts, B. Mennucci, H. P. Hratchian, J. V. Ortiz, A. F. Izmaylov, J. L. Sonnenberg, D. Williams-Young, F. Ding, F. Lipparini, F. Egidi, J. Goings, B. Peng, A. Petrone, T. Henderson, D. Ranasinghe, V. G. Zakrzewski, J. Gao, N. Rega, G. Zheng, W. Liang, M. Hada, M. Ehara, K. Toyota, R. Fukuda, J. Hasegawa, M. Ishida, T. Nakajima, Y. Honda, O. Kitao, H. Nakai, T. Vreven, K. Throssell, J. A. Montgomery, Jr., J. E. Peralta, F. Ogliaro, M. Bearpark, J. J. Heyd, E. Brothers, K. N. Kudin, V. N. Staroverov, T. Keith, R. Kobayashi, J. Normand, K. Raghavachari, A. Rendell, J. C. Burant, S. S. Iyengar, J. Tomasi, M. Cossi, J. M. Millam, M. Klene, C. Adamo, R. Cammi, J. W. Ochterski, R. L. Martin, K. Morokuma, O. Farkas, J. B. Foresman, and D. J. Fox, Gaussian 09, Revision A.02, Gaussian, Inc., Wallingford CT, 2016.

[S2] T. Michinobu, J. C. May, J. H. Lim, C. Boudon, J.-P. Gisselbrecht, P. Seiler, M. Gross, I. Biaggio and F. Diederich, *Chem. Commun.*, **2005**, 737-739.

[S3] M. Kivala, C. Boudon, J.-P. Gisselbrecht, P. Seiler, M. Gross and F. Diederich, *Chem. Commun.*, **2007**, 4731-4733.

[S4] X. Tang, W. Liu, J. Wu, C.-S. Lee, J. You and P. Wang, *J. Org. Chem.*, **2010**, 75, 7273-7278.

[S5] W. Humphrey, A. Dalke, K. Schulten, *J. mol. graph.* **1996,** *14*, 33-38

[S6] Y. Niu, W. Li, Q. Peng, H. Geng, Y. Yi, L. Wang, G. Nan, D. Wang, Z. Shuai, *Mol. Phys.* **2018,** *116*, 1078-1090.

[S7] K. A. Leonard, M. I. Nelen, L. T. Anderson, S. L. Gibson, R. Hilf and M. R. Detty, *J. Med. Chem.*, **1999**, *42*, 3942-3952.
